# Supplementary material for: Design and high-throughput implementation of MALDI-TOF/MS-based assays for Parkin E3 ligase activity
Source: Cell Rep Methods. 2024 Feb 20;4(2):100712. doi: 10.1016/j.crmeth.2024.100712 (PMC10921019; doi:10.1016/j.crmeth.2024.100712)
Supplement: Document S1. Figures S1–S5 [file mmc1.pdf]

Cell Reports Methods, Volume *4*

Supplemental information

Design and high-throughput implementation of MALDI-TOF/MS-based assays for Parkin E3 ligase activity

Ryan Traynor, Jennifer Moran, Michael Stevens, Odetta Antico, Axel Knebel, Bahareh Behrouz, Kalpana Merchant, C. James Hastie, Paul Davies, Miratul M.K. Muqit, and Virginia De Cesare

Supplementary Figures

Sup. Figure 1

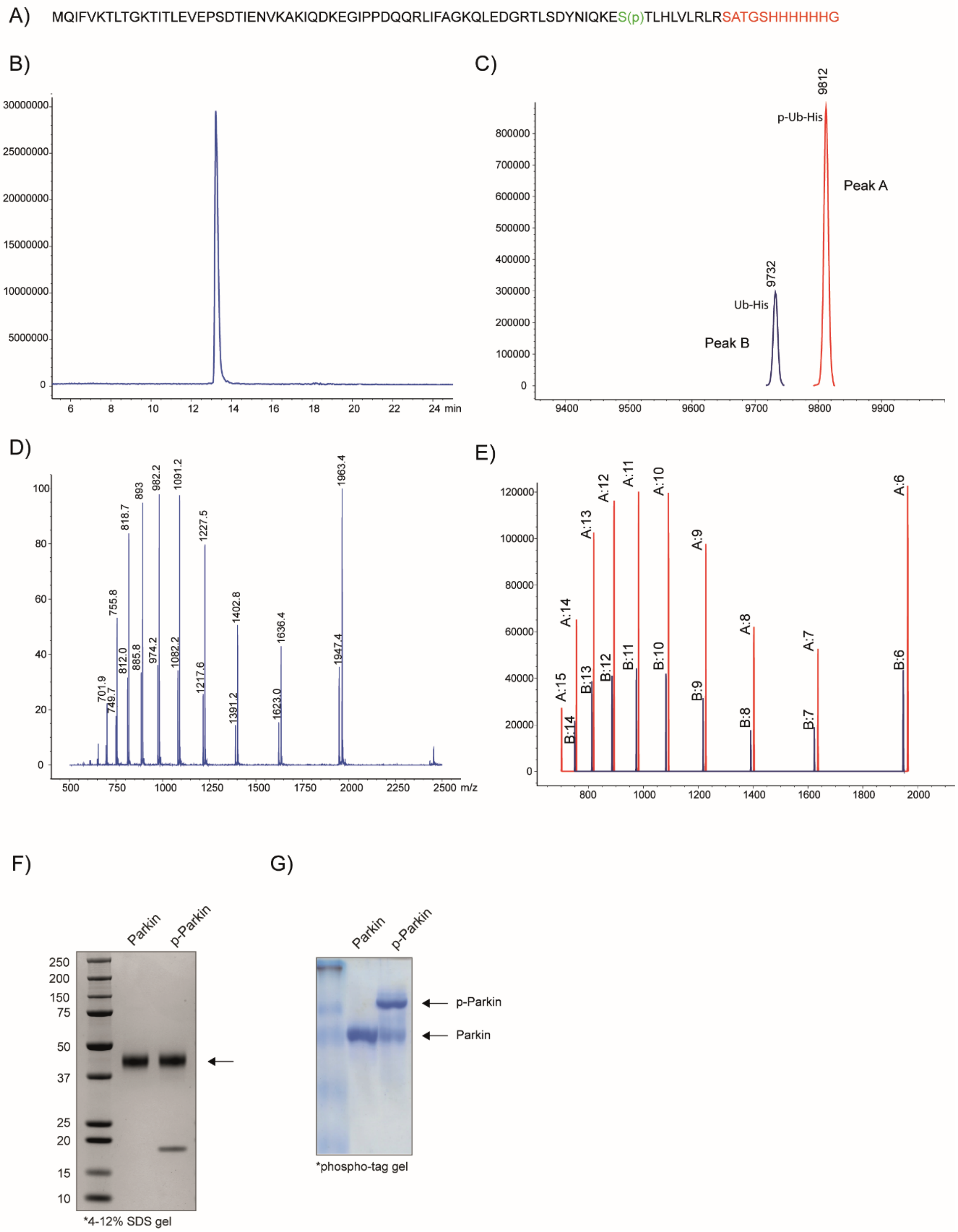

A) MQIFVKTLTGKTITLEVEPSDTIENVKAKIQDKEGIPPDQQRLIFAGKQLEDGRTLSDYNIQKES(p)TLHLVLRLRSATGSHHHHHHG
B)
C)
9812
p-Ub-His
Peak A
9732
Ub-His
Peak B
D)
E)
F)
Parkin
p-Parkin
*4-12% SDS gel
G)
Parkin
p-Parkin
p-Parkin
Parkin
*phospho-tag gel

Sup Figure 1|His tagged Phosphorylated Ubiquitin (p-Ub-His), Parkin and pParkin Quality Control (related to Figure 2). (A) p-Ub-His protein sequence: serine 65 (green) and C-terminal 6His tag (red). p-Ub-His Quality Control (QC) performed via Liquid Chromatography - Mass Spectrometry (LC-MS) analysis. (B) LC-MS chromatogram of p-Ub-His. (C) p-Ub-His MS components: 9812 *m/z* peak, corresponding to the p-Ub-His expected *m/z*, and 9732 *m/z* peak corresponding to the remaining not phosphorylated counterpart. Purity level have been considered into experimental calculations. (D) p-Ub-His QC Mass Spectrum. (E) p-Ub-His QC Deconvoluted Ion Set. (F) Parkin and p-Parkin purity check by SDS-page. (G) Parkin phosphorylation efficiency: phospho-tag gels indicates that p-Parkin is about 70% pure. Arrow indicates main WT or p-Parkin species.

Sup Fig. 2

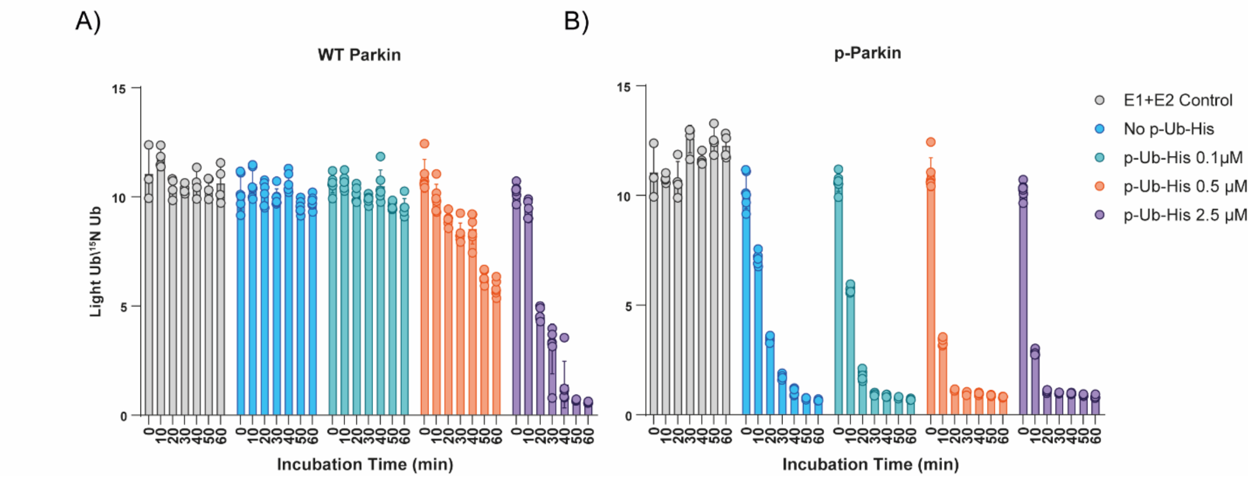

Sup. Figure 2| MALDI-TOF Ubiquitylation assay: Light/^{15}N ubiquitin ratio (related to Figure 2). Data shown before conversion into Residual Activity%. (A) WT Parkin MALDI-TOF autoubiquitylation assay: stable level of light/^{15}N Ub in the E1+E2 control indicates no consumption of ubiquitin in presence of the E1 activating enzyme and UBE2L3 conjugating enzyme only. (B) Corresponding dataset for p-Parkin MALDI-TOF autoubiquitylation assay. Data are represented as mean ± SD.

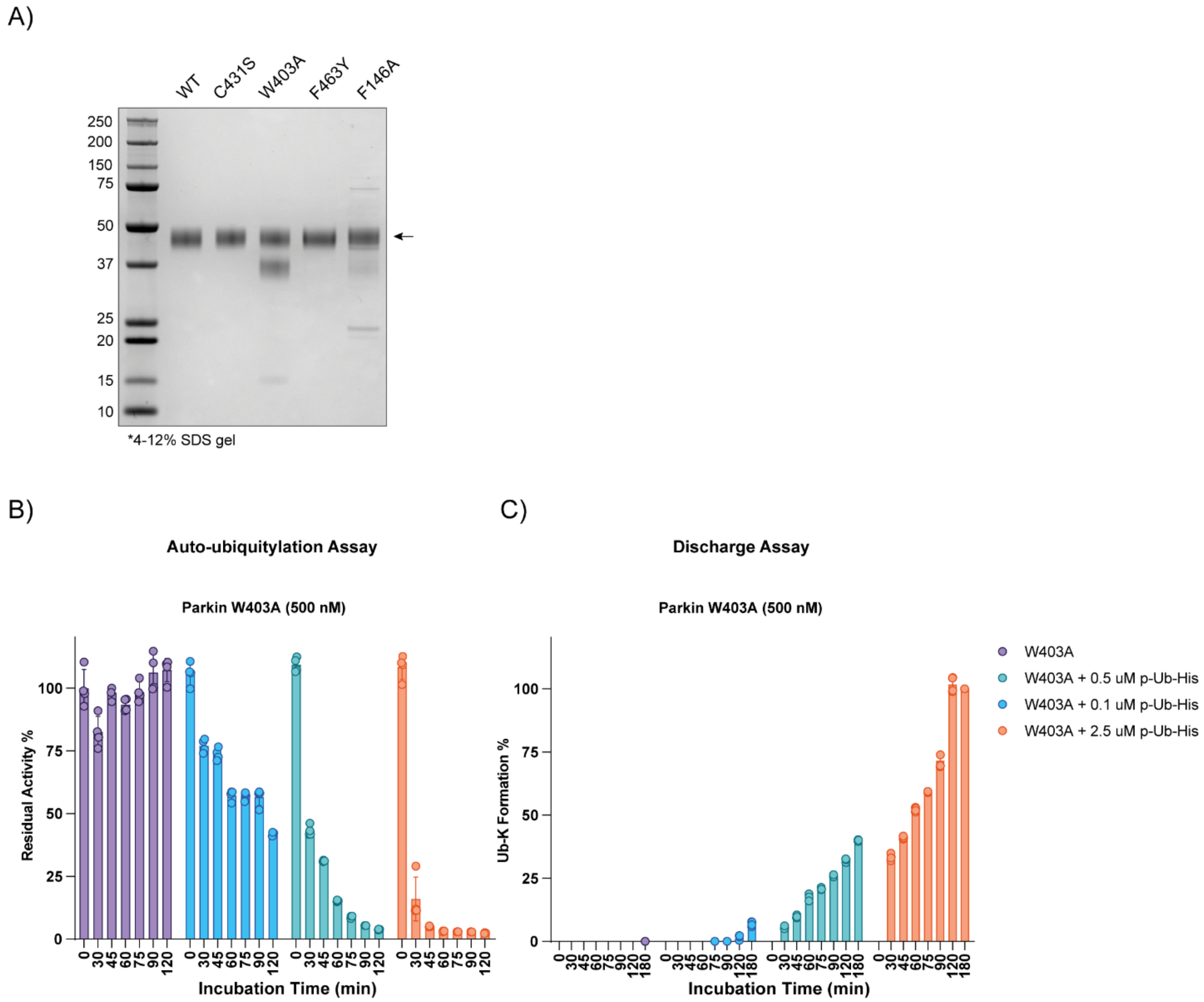

Sup. Figure 3| Parkin activating mutants purity check by SDS-page and activity at 500 nM (related to Figure 3). (A) Parkin WT, C431S, W403A, F463A and F146A were expressed and purified as indicated in Material and Methods. Relative quantification (main band indicated by the arrow) was implemented to account for the presence of impurities. (B) Activity of Parkin W403A was assessed at 500nM via MALDI-TOF autoubiquitylation assay. (C) Activity of Parkin W403A was assessed at 500nM via MALDI-TOF discharge assay. No background activity was detected when testing Parkin W403A at the final concentration of 500nM in absence of p-Ub-His. Indicated amount of p-Ub-His efficiently activated Parkin W403A. Data are represented as mean ± SD

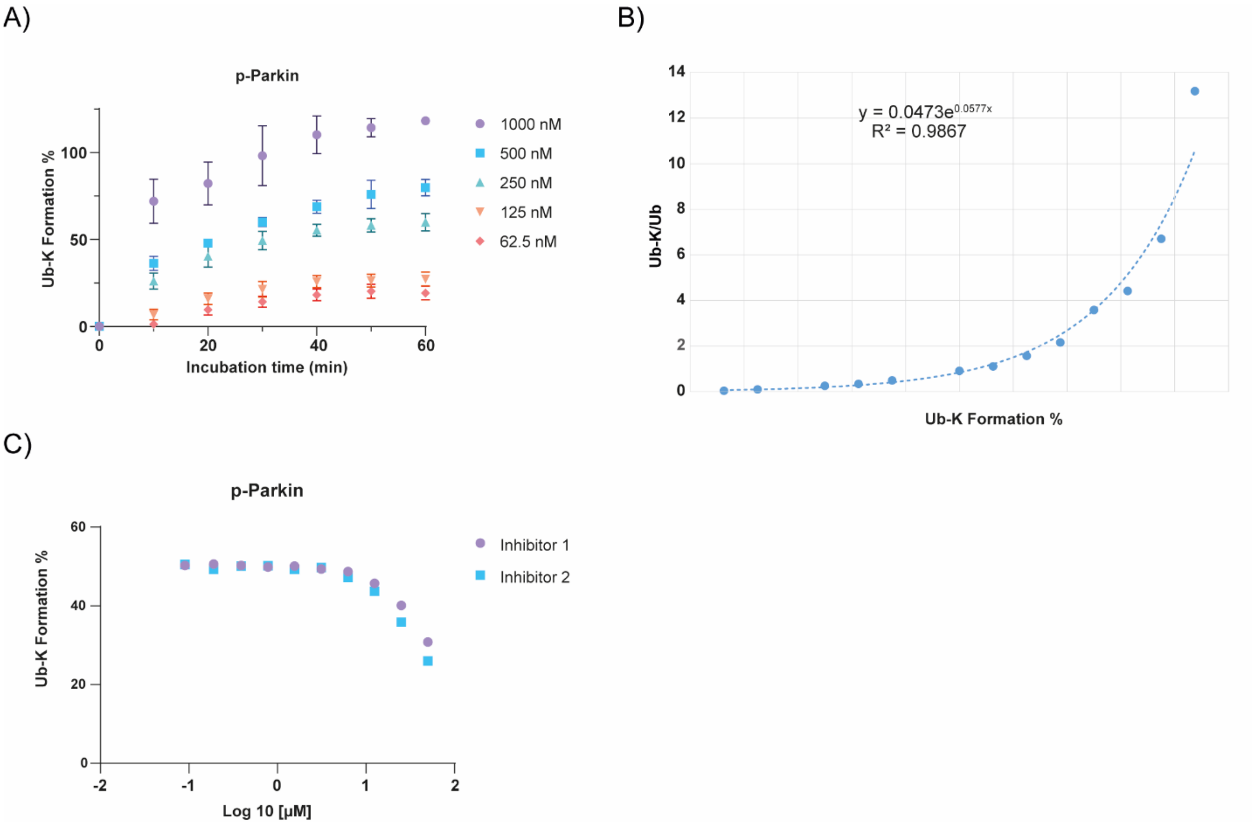

Sup. Figure 4| Identification of HTS conditions for p-Parkin MALDI-TOF discharge assay (related to Figure 4). (A) p-Parkin was incubated at 25° at the indicated at concentrations, and Ub-K formation was followed over a time course experiment. Data are represented as mean ± SD. (B) Linearity curve for Ub-K formation %. Known amounts of Ub (substrate) and Ub-K (product) were mixed and analysed by MALDI-TOF/MS. Resulting curve and associated exponential equation was employed to translate Ub-K/Ub peak area ratio into Ub-K Formation%. Data are represented as average of two datapoints. (C) Validation of two p-Parkin inhibitors identified via HTS. Compound 1 and compound 2 were tested at the indicated concentrations. Both compound 1 and compound 2 reduced p-Parkin activity at 10 µM, 25 µM and 50 µM. Data are represented as average of two datapoints.

Sup Fig. 5

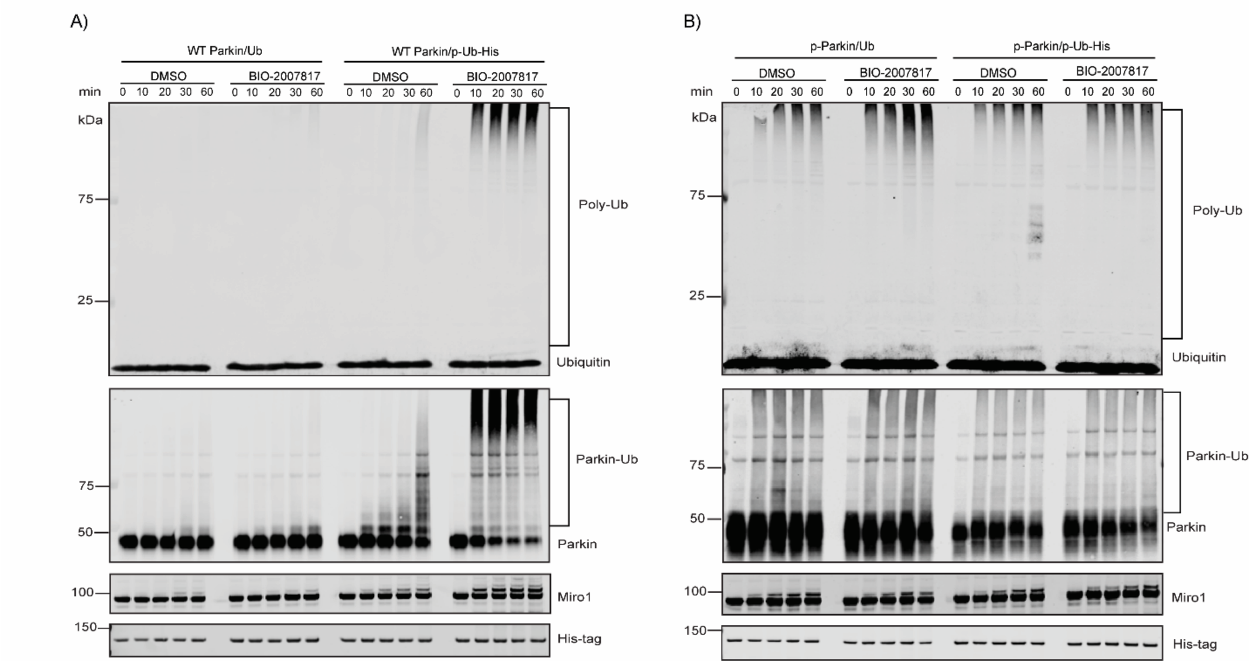

Sup. Fig. 5| Validation of BIO-2007817 as WT and p-Parkin activators via Western Blot analysis (related to Figure 4). (A) WT Parkin was tested in presence of BIO2007817 (100 µM) or DMSO (Control) with ubiquitin only (Ub) and with Ub + stoichiometric amount of p-Ub-His (relative to WT or p-Parkin). The reaction was stopped at the indicated time points and samples were run on SDS-page. Parkin and Miro1 ubiquitylation species were detected by Western Blot. (B) Similar to panel A, p-Parkin was tested in presence of BIO2007817 (100 µM) or DMSO (Control) with ubiquitin only (Ub) and with Ub + stoichiometric amount of p-Ub-His (relative to p-Parkin).
