# Supplementary material for: Design and high-throughput implementation of MALDI-TOF/MS-based assays for Parkin E3 ligase activity
Source: Cell Rep Methods. 2024 Feb 20;4(2):100712. doi: 10.1016/j.crmeth.2024.100712 (PMC10921019; doi:10.1016/j.crmeth.2024.100712)
Supplement: Document S2. Article plus supplemental information [file mmc2.pdf]

Cell Reports Methods

Report

Design and high-throughput implementation of MALDI-TOF/MS-based assays for Parkin E3 ligase activity

Graphical abstract

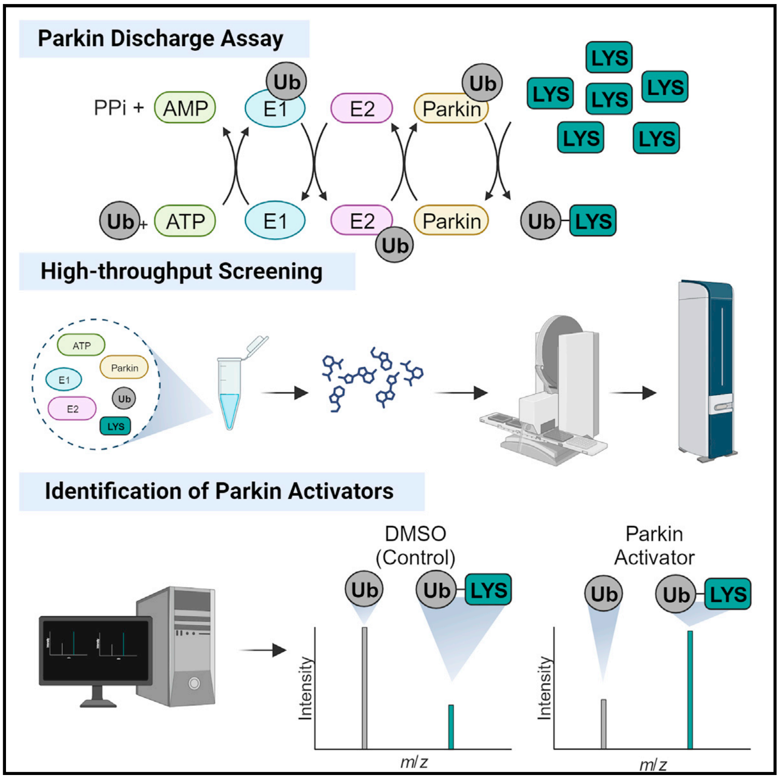

Highlights

- Development of MALDI-TOF/MS-based assays for the assessment of Parkin activity
- Accurate quantification of the impact of point mutations on Parkin's activity
- Implementation of the MALDI-TOF discharge assay for high-throughput screening purposes
- Validation of previously reported Parkin activators

Authors

Ryan Traynor, Jennifer Moran, Michael Stevens, ..., Paul Davies, Miratul M.K. Muqit, Virginia De Cesare

Correspondence

Vdecesare@dundee.ac.uk

In brief

Traynor et al. present two MALDI-TOF/MS-based assays for quantifying Parkin activity *in vitro*. The assays provide precise E3 ligase activity measurements by leveraging Parkin-mediated auto-ubiquitylation and discharge on lysine residues. A proof-of-concept high-throughput screening and the validation of Parkin activators showcase assay robustness and sensitivity, providing a valuable addition to Parkinson's disease research.

Traynor et al., 2024, Cell Reports Methods *4*, 100712
February 26, 2024 Crown Copyright © 2024
https://doi.org/10.1016/j.crmeth.2024.100712

CellPress

Cell Reports Methods

CellPress OPEN ACCESS

Report

Design and high-throughput implementation of MALDI-TOF/MS-based assays for Parkin E3 ligase activity

Ryan Traynor,[1] Jennifer Moran,[1] Michael Stevens,[1] Odetta Antico,[1] Axel Knebel,[1] Bahareh Behrouz,[2] Kalpana Merchant,[3] C. James Hastie,[4] Paul Davies,[1] Miratul M.K. Muqit,[1] and Virginia De Cesare[1,5,*]
[1]MRC Protein Phosphorylation and Ubiquitylation Unit, School of Life Sciences, University of Dundee, Dow St, Dundee DD1 5EH, Scotland, UK
[2]Vincere Biosciences, Inc., 245 Main St. Fl 2, Cambridge, MA 02142, USA
[3]Northwestern University Feinberg School of Medicine, Chicago, IL 60611, USA
[4]MRC Protein Phosphorylation and Ubiquitylation Unit Reagents and Services, School of Life Sciences, University of Dundee, Dow St., Dundee DD1 5EH, Scotland, UK
[5]Lead contact
*Correspondence: Vdecesare@dundee.ac.uk
https://doi.org/10.1016/j.crmeth.2024.100712

MOTIVATION Parkinson's disease lacks a definitive pharmacological cure. Enhancing the activity of the E3 ligase Parkin holds promise for treating this progressive neurological condition. Yet, limited availability of high-throughput *in vitro* assays hinders the discovery of therapeutic molecules targeting Parkin. Here, we introduce two MALDI-TOF/MS-based, label-free, and sensitive approaches, designed to identify Parkin activators. This method responds to the pressing need for more effective drug discovery campaigns in Parkinson's research and provides complementary tools for undertaking primary high-throughput screening for the identification of Parkin modulators.

SUMMARY

Parkinson's disease (PD) is a progressive neurological disorder that manifests clinically as alterations in movement as well as multiple non-motor symptoms including but not limited to cognitive and autonomic abnormalities. Loss-of-function mutations in the gene encoding the ubiquitin E3 ligase Parkin are causal for familial and juvenile PD. Among several therapeutic approaches being explored to treat or improve the prognosis of patients with PD, the use of small molecules able to reinstate or boost Parkin activity represents a potential pharmacological treatment strategy. A major barrier is the lack of high-throughput platforms for the robust and accurate quantification of Parkin activity *in vitro*. Here, we present two different and complementary Matrix-Assisted Laser Desorption/Ionization Time-Of-Flight Mass Spectrometry (MALDI-TOF/MS)-based approaches for the quantification of Parkin E3 ligase activity *in vitro*. Both approaches are scalable for high-throughput primary screening to facilitate the identification of Parkin modulators.

INTRODUCTION

The coordinate action of the really interesting new gene (RING)-IBR-RING (RBR) E3 ubiquitin (Ub) ligase Parkin and PTEN-induced kinase 1 (PINK1) is fundamental for the clearance of dysfunctional mitochondria by mitophagy in nearly every cell type including neurons.[1–3] Under healthy cellular conditions, Parkin is present in the cytosol in an auto-inhibited conformation.[4–6] Upon mitochondrial depolarization, which can be induced by mitochondrial uncouplers, PINK1 is activated and recruits and activates Parkin at sites of mitochondrial damage via directly phosphorylating Parkin at serine 65 (S65) within its Ub-like domain (p-Parkin) and indirectly by phosphorylating Ub (p-Ub), also on serine 65.[7–9] Under *in vitro* assay conditions of Parkin activation, Parkin and p-Parkin undergo auto-ubiquitylation on accessible lysine residues, can catalyze Ub transfer to substrates, or can stimulate discharge of Ub from a charged E2 enzyme, UBE2L3, onto primary amines present in the reaction buffer (discharge assay). Both types of ubiquitylation have previously been used as readouts for Parkin and p-Parkin activity.[9] Low-throughput, SDS-PAGE-based-techniques have been extensively applied for visualizing Parkin auto-ubiquitylation patterns.[9,10] Ub-fluorescent probes have also been developed to exploit the reactivity of Parkin toward primary amines.[11]

This is an open access article under the CC BY license (http://creativecommons.org/licenses/by/4.0/).

While both these approaches were shown to be easy tools for investigating Parkin activity *in vitro*, they have substantial caveats and limitations. SDS-PAGE-based techniques lack scalability to high-throughput format and are often not fully quantitative, while fluorescent based-approaches are intrinsically prone to fluorescence-related artifacts. Here, we described two quantitative and complementary Matrix-Assisted Laser Desorption/Ionization Time-Of-Flight Mass Spectrometry (MALDI-TOF/MS)-based assays to determine Parkin and p-Parkin activity *in vitro*. Both methods allow for quantitative investigation of Parkin activity *in vitro*, are scalable to high-throughput formats, and employ physiological substrates (Ub and p-Ub), thus circumventing artifacts associated with the use of fluorescence-based tools.

RESULTS

Development of MALDI-TOF/MS-based Parkin activity assays

We previously reported the development of a MALDI-TOF/MS-based method for the quantification of the activity of E2-conjugating enzymes and E3 ligases belonging to the RING, HECT, and RBR families[12,13] (named the MALDI-TOF E2/E3 assay or MALDI-TOF autoubiquitylation assay). While RING E3 ligases rely on the catalytic activity of a cognate E2-conjugating enzyme, HECT and RBR E3 ligases receive Ub from the E2-conjugating enzymes to ubiquitylate their substrate on lysine residues. Most E3 ligases will undergo auto-ubiquitylation when tested *in vitro*. The previously published MALDI-TOF autoubiquitylation assay was based on quantification of the progressive disappearance of Ub as a consequence of its utilization in the auto-ubiquitylation process.[12] Since the reactivity toward lysine is mediated by the E3 ligase in the RBR enzymatic cascade, we explored whether we could determine Parkin reactivity using a complementary lysine discharge method (also known as the nucleophile reactivity assay[14]) followed by MALDI-TOF/MS detection, named MALDI-TOF discharge assay. Both MALDI-TOF/MS-based assays utilize unlabeled ubiquitin as substrate and untagged (His-SUMO-cleaved) recombinant human Parkin expressed in *Escherichia coli* as previously described.[15] UBE2L3 is a HECT-RBR-specific E2-conjugating enzyme that lacks intrinsic E3-independent reactivity toward lysine residues.[14] Therefore, the ability to discharge on lysine—and the consequent formation of Ub-lysine adducts (Ub-K)—relies exclusively on the activity of a cognate HECT or RBR E3 ligase. In the MALDI-TOF auto-ubiquitylation assay, quantification of Parkin activity is achieved by comparing the signal of Ub to that of the heavy-labeled Ub internal standard (^{15}N Ub). Therefore, the auto-ubiquitylation rate can be represented as a linear reduction of detectable Ub over time (residual Ub %) (Figure 1A). In contrast, in the MALDI-TOF discharge assay, both the substrate (Ub) and the Ub-K product change over time as the former is converted to the latter. Consequently, the mathematical representation of the discharge assay method will be a non-linear function, as both substrate and product measurements change over time (Figure 1B). Therefore, in the MALDI-TOF discharge assay, a dedicated standard curve must be defined in advance to determine the rate of product formation (Ub-K formation %) (Figure 1B). The unique regulation of wild-type (WT) Parkin requires the combined use of Ub and p-Ub. The interaction between p-Ub and Parkin releases Parkin's auto-inhibitory state; therefore, p-Ub functions as an allosteric Parkin modulator. Due to the closeness in molecular weight between p-Ub (8,646.7 *m/z*) and the ^{15}N Ub internal standard (8,669.7 *m/z* observed), we employed His$_6$-tagged p-Ub (p-Ub-His; 9,812 *m/z*; Figures S1A–S1E) to prevent interference with the ^{15}N Ub signal. The His$_6$ tag present at the C terminus of p-Ub-His and the absence of a final glycine dyad do not allow for the incorporation of p-Ub-His into poly-Ub chains. The MALDI-TOF auto-ubiquitylation assay exhibited slower kinetics compared to the MALDI-TOF discharge assay. This can be attributed to the structural rearrangements Parkin must undergo for auto-ubiquitylation, whether in *cis* or in *trans* mode, and the limited availability of lysine residues for Ub conjugation. In contrast, the MALDI-TOF discharge assay benefits from a high concentration of Ac-K (50 mM), which provides an abundant supply of reactive sites for Ub, facilitating the rapid formation of Ub-K adducts. Therefore, to achieve comparable reaction rates, the MALDI-TOF auto-ubiquitylation assay was performed at 37°C, while the discharge assay was performed at room temperature.

Inherent differences in experimental settings between the MALDI-TOF auto-ubiquitylation and discharge assays do not allow for the direct comparison of their readout. However, results obtained from both assays closely align with each other, providing consistent observations despite the differences in kinetics.

Assessing Parkin activity by MALDI-TOF auto-ubiquitylation and discharge assay

The activity of Parkin is tightly regulated both by direct phosphorylation and by the interaction with p-Ub.[2,9] We employed the MALDI-TOF auto-ubiquitylation and discharge assays to accurately quantify the contribution of these regulatory layers on Parkin activity rate. In the MALDI-TOF auto-ubiquitylation assay, Parkin activity was quantified by the progressive reduction of the mono-Ub peak, while in the discharge assay, Parkin activity was assessed by the formation of Ub-Ac-K product. Both MALDI-TOF/MS-based methods were employed to quantify the activity of Parkin and p-Parkin (expressed as previously described[15]; Figures S1F and S1G) upon addition of increasing amount of p-Ub-His. WT Parkin and p-Parkin were tested at a final concentration of 250 nM. Reactions were started by the addition of Ub supplemented with three different concentrations of p-Ubi-His: 100, 500, and 2500 nM. In the auto-ubiquitylation assay, data were firstly normalized over the ^{15}N Ub internal standard signal (light Ub/^{15}N Ub), and a control reaction without Parkin present (E1+E2 control) was used to establish the rate of Parkin-dependent Ub consumption (Figures S2A and S2B). We found that an amount of p-Ub-His stoichiometrically equivalent to WT Parkin (500 nM) is sufficient to partially activate WT Parkin (Figure 2A), while 5 times excess of p-Ub-His induced WT Parkin activity levels comparable to those of p-Parkin in the absence of p-Ub-His (Figure 2A). Stoichiometric amounts of p-Ub-His double the auto-ubiquitylation rate of p-Parkin after 10 min (residual

CellPress
OPEN ACCESS

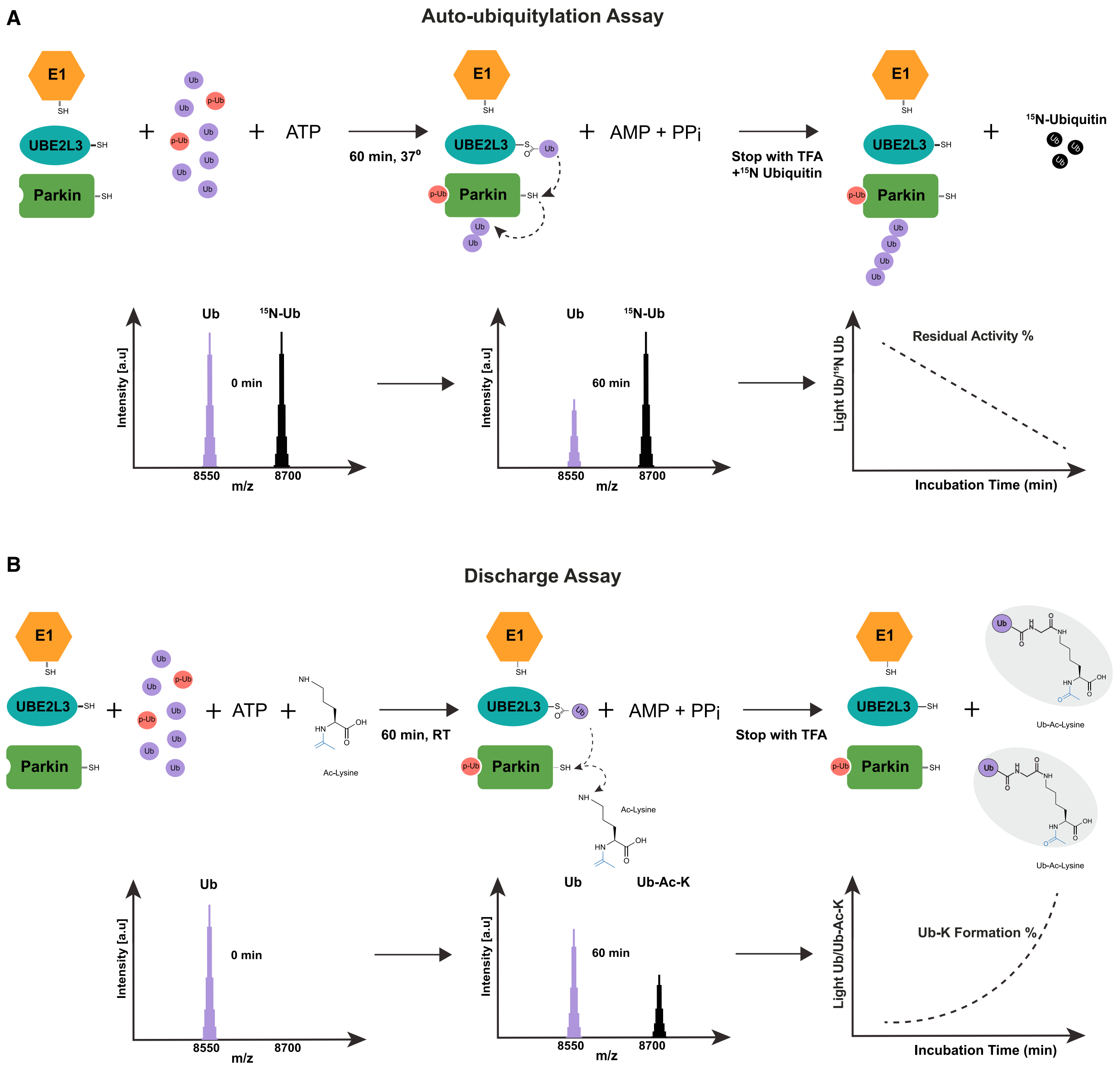

Figure 1. Schematic representation of MALDI-TOF/MS Parkin auto-ubiquitylation and discharge assay

(A) Parkin auto-ubiquitylation reduces the pool of Ub detected by MALDI-TOF/MS over time. Quantification is achieved by use of ^{15}N Ub as internal standard consistently present in the reaction (light Ub/^{15}N Ub).

(B) Parkin-dependent formation of Ub-Ac-lysine (Ub-K) is detected by MALDI-TOF/MS. Quantification is achieved by measuring the ratio between the substrate (Ub) and the product (Ub-K). A linearity curve allows us to translate Ub/Ub-K ratio into the percentage of Ub-K formation.

Ub 66% in the absence of p-Ub-His compared to 31.5% in the presence of 500 nM p-Ub). In the discharge assay, WT Parkin is efficiently activated by stoichiometric amounts of p-Ub-His. A similar effect was observed for p-Parkin, whose activity is greatly enhanced already in the presence of sub-stoichiometric amounts of p-Ub-His (Ub-K formation 22% in the absence of p-Ub compared to 78% in the presence of 100 nM p-Ub) (Figure 2B). Phospho-tag SDS-gel analysis indicated that about 70% of Parkin was phosphorylated (Figure S1G); therefore, when testing p-Parkin in the presence of p-Ub-His, it is not possible to discriminate whether the increase in activity is due to the activation of WT Parkin compared or the overactivation of p-Parkin. Overall, both MALDI-TOF-based assays accurately and quantitively measured the E3 ligase activity of Parkin and p-Parkin and the rate at which the co-factor p-Ub-His activates WT Parkin and may further activate p-Parkin.

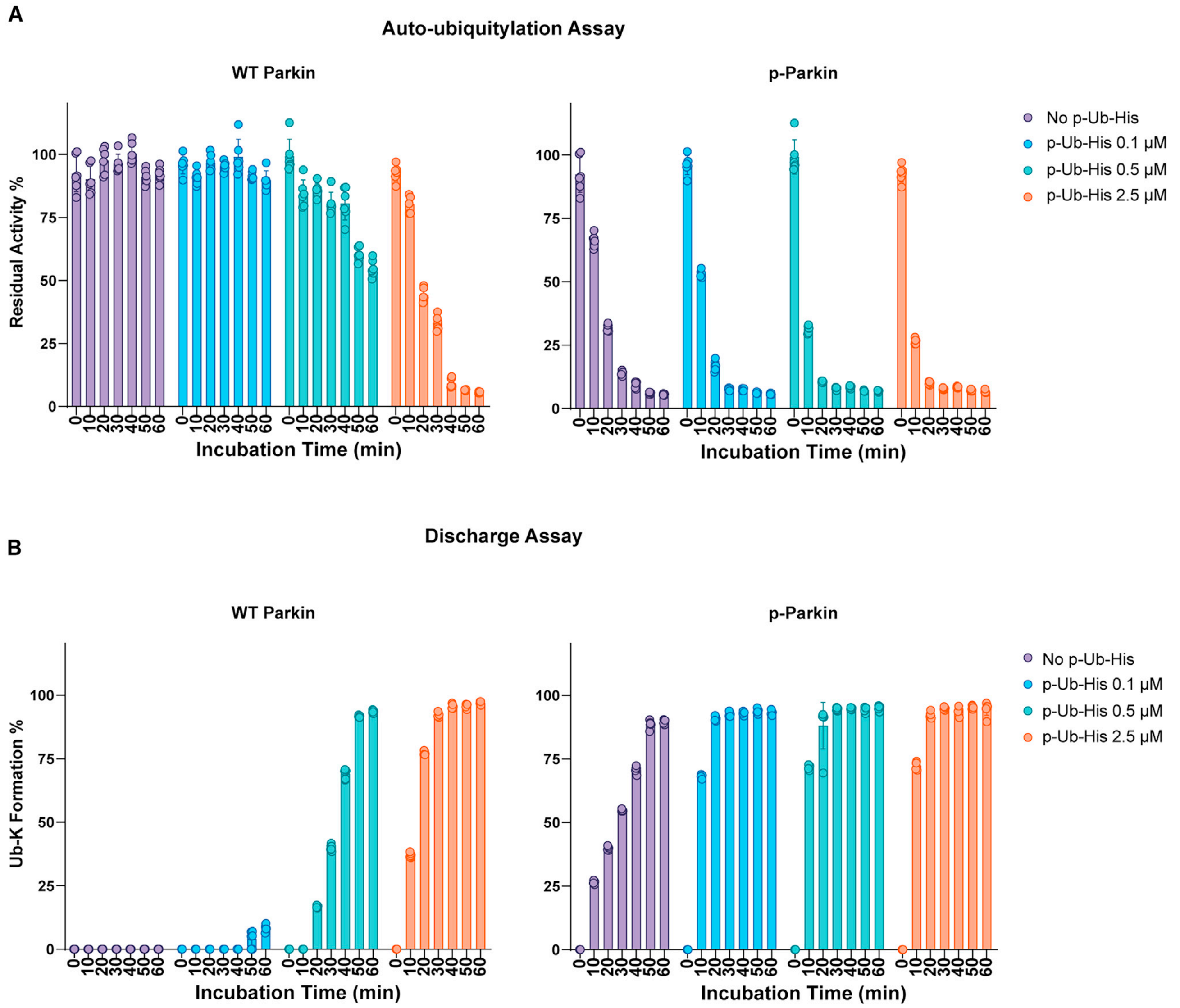

Figure 2. Quantification of WT Parkin and p-Parkin activity by MALDI-TOF auto-ubiquitylation and discharge assay

(A) WT Parkin and p-Parkin were incubated in the absence or presence of increasing amounts of p-Ub-His for up to 60 min. The reduction of mono-Ub as a consequence of WT Parkin and p-Parkin activity is reported as Residual Activity %.

(B) WT Parkin and p-Parkin were incubated in the absence or presence of increasing amounts of p-Ub-His for up to 60 min. The Ub-K % formation rate indicates activity in the MALDI-TOF discharge assay readout. Data points are representative of at least 3 replicates ±SD.

Quantifying the effect of point mutations on Parkin activity

Structural analysis of inactive and active Parkin has identified three major interdomain interfaces that maintain auto-inhibition of Parkin Ub ligase activity.[5,6,16] Based on these studies, the point mutation W403A disrupts the interaction between the repressor element of Parkin (REP) and the RING1 domain. Similarly, introducing F146A or F463Y point mutations loosens the interaction between REP and the RING0-RING2 interface. Each of these point mutations release the auto-inhibitory conformation of Parkin and are effective at promoting Parkin activity[5,6,17–19] as well as rescuing defects in p-Ub binding and S65 phosphorylation.[18] We therefore expressed Parkin W403A, F463Y, and F146A mutants as well as the catalytically inactive C431S mutant (Figure S3A) and compared the impact of these mutations on Parkin activation using both the MALDI-TOF auto-ubiquitylation and discharge assays. Since Parkin-activating-mutants only partially release E3 ligase activity, enzymatic concentrations and incubation times were optimized. The activity of W403A Parkin could not be detected in the absence of p-Ub-His at the concentration of 500 nM (Figures S3B and S3C). The lack of W403A activity at a low concentration and in the absence of the co-factor p-Ub-His is due to the relatively low level of activity released by this point mutation.

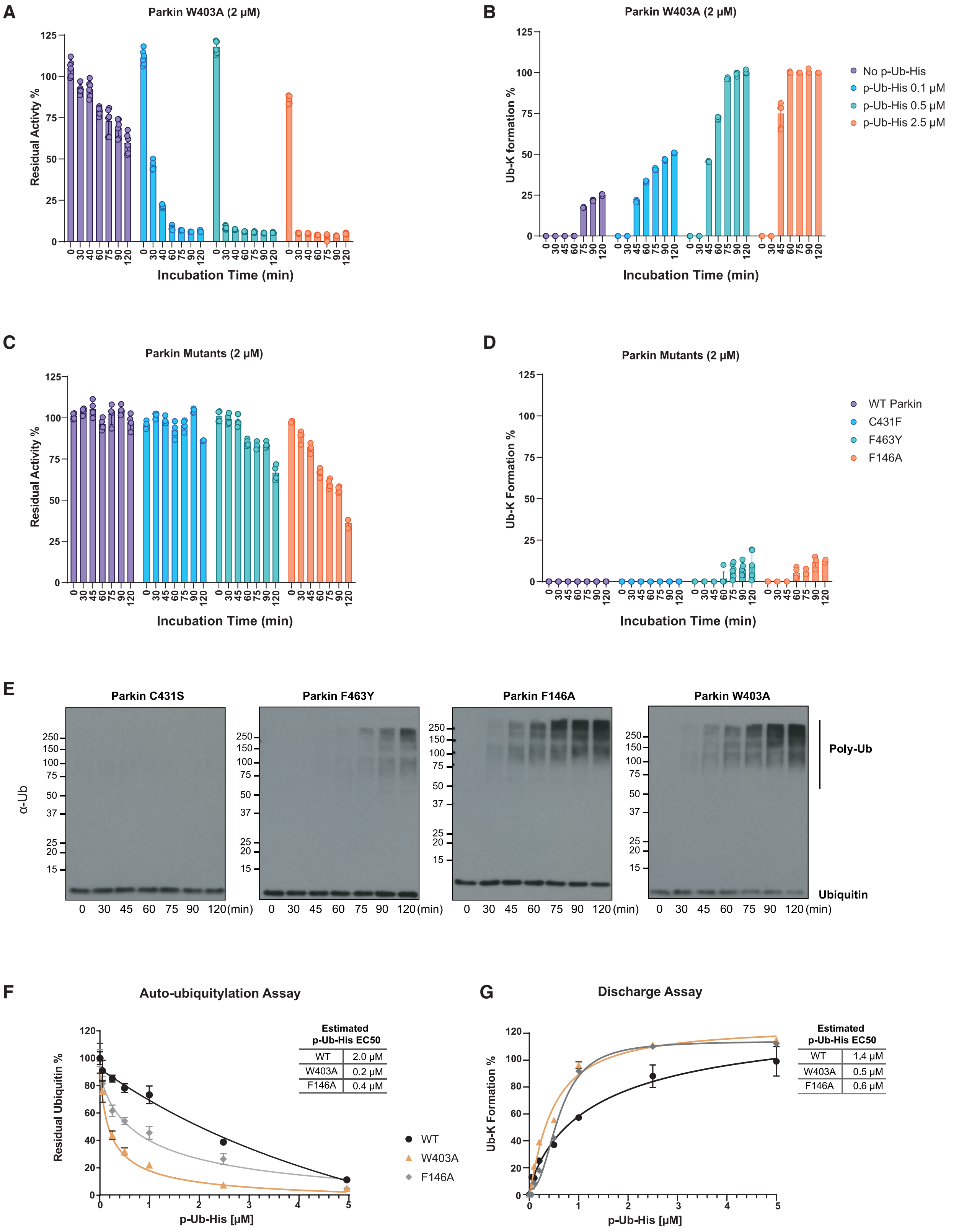

(legend on next page)

By contrast, Parkin W403A activity was detected at the final concentration of 2 μM using both the MALDI-TOF auto-ubiquitylation assay (Figure 3A) and the MALDI-TOF discharge assay (Figure 3B). Note that reaction rates were captured over an extended time frame of up to 120 min. W403A background auto-ubiquitylation activity (in the absence of p-Ub-His) reduces the initial Ub pool after 120 min of incubation by ~40% (Figure 3A), and reaction rates are further increased by the addition of increasing amounts of p-Ub-His (Figures 3A and 3B). The F146A mutant showed a level of activity comparable to that of W403A, with only 36.2% of the initial pool of Ub still detectable after 120 min (Figure 3C). The active mutant F463Y showed ~50% less activity compared to W403A and F146A mutants (~70% of Ub still present after 120 min; Figure 3C). A similar trend was observed in the discharge assay (Figure 3D), albeit the measured activity of the Parkin mutants in the discharge assay were relativity lower compared to the auto-ubiquitylation assay. This likely reflects the distinct temperature at which respective assays were performed. We confirmed these findings using an orthogonal Parkin *in vitro* assay in which mutant Parkin F463Y, F164A, and W403A and C431F were incubated in the presence of adenosine triphosphate (ATP), $MgCl_2$, E1 Ub-activating enzyme, UBE2L3-conjugating enzyme, and Ub. After 60 min, reactions were terminated with SDS sample buffer in the presence of 2-mercaptoethanol and heated to 100°C, and ubiquitylation was assessed by immunoblot analysis with antibodies that detect Ub (Figure 3E). We further employed the MALDI-TOF-based assays to estimate the half-maximal effective concentration (EC50) of p-Ub-His for the activation of WT, W403A, and F146A Parkin. We incubated 500 nM WT, W430A, and F146A Parkin with increasing concentrations of p-Ub-His (0.05, 0.2, 0.5, 1, 2.5, and 5 μM) and incubated the reaction 30 min in the previously defined conditions. An estimated EC50 of 2 μM for WT Parkin, 0.2 μM for W403A, and 0.4 μM for F146A was determined in the MALDI-TOF auto-ubiquitylation assay settings (Figure 3F). A similar trend was observed for the MALDI-TOF discharge assay: 1.4 μM for WT Parkin, 0.5 μM for W403A, and 0.6 μM for F146A (Figure 3G). The results confirmed that both W403A and F146A Parkin mutants require reduced amounts of p-Ub-His to achieve activity levels comparable to those of WT Parkin. Both assays indicate that the W403A mutation requires between 2- and 10-fold less p-Ub-His to achieve WT Parkin activity levels. Overall, our analysis of Parkin mutants is consistent with the previous literature reporting W403A as one of the most activating Parkin single-point mutations.[5,6,16] Moreover, the accurate quantification of the absolute and relative activation effect of Parkin point mutations further validates the ability of both MALDI-TOF-based assays to identify Parkin activation and inhibition rates.

Development of Parkin high-throughput screen

Primary, activity-based high-throughput screening (HTS) represents often the first step when starting a new drug discovery project that targets an enzyme. Such a step is fundamental for the identification of promising candidates from the vast number of natural and synthetic compound libraries available. Since PD is caused by loss of function of Parkin,[20,21] the pharmaceutical intent is to re-instate the enzymatic activity of Parkin through the identification of Parkin-specific activators.[22] The MALDI-TOF auto-ubiquitylation assay relies on the progressive reduction of the Ub signal: potential Parkin activators might accelerate the disappearance of Ub to an extent where no free Ub will be detected. This limits the assay window, which, in turn, negatively impacts the Z' score calculation and limits the identification of potential activators and the quantification of activation rates in dose-response experiments. Also, the necessity of an internal standard (^{15}N Ub) relies on the precision of a further liquid-dispensing step, which impacts on the overall signal variability and Z' score levels. On the other hand, the MALDI-TOF discharge assay offers a larger assay window and the possibility to work at lower temperatures (25°C) and without using the ^{15}N Ub as internal standard. Therefore, we tested the feasibility of employing the MALDI-TOF discharge assay to perform a preliminary HTS for the identification of p-Parkin activators. To optimize the HTS conditions, various amounts of p-Parkin ranging from 62.5 nM to 1 μM were tested over a time course experiment (Figure S4A). Among these trials, the reaction containing 250 nM p-Parkin, which was halted at the 20 min mark, proved to be the most suitable condition for the HTS settings. We therefore tested a library of about 20,000 compounds predicted to be able to permeate the blood-brain barrier. The HTS workflow was designed to be scalable and adaptable for an HTS campaign and consists of 3 steps: preincubation of 5 μL enzymatic mixture with compounds (10 μM in 100% DMSO), reaction initiation by adding 5 μL substrate (Ub and 50 mM Ac-K), and reaction termination with 5 μL 6% TFA (Figure 4A). The use of high-density 1536 AnchorChip MALDI targets allowed us to combine up to four 384 assay plates into one MALDI-TOF/MS run (Figure 4A). Each plate included a column (16 wells) reserved for positive controls (no compound present, only DMSO) and one column for negative controls (reaction in the absence of p-Parkin, where only background reading should be detected; example data are shown in Figure 4B). Data were normalized by dividing the area of the substrate (Ub) by the area of the product (Ub-Ac-K). A linearity curve with known amounts of Ub and Ub-Ac-K was interpolated and used to translate the Ub-Ac-K/Ub ratio into the percentage of Ub-K formation (Figure S4B). The robustness of HTS is a function of both the variability of positive and negative controls and the statistical space for the robust identification of

Figure 3. Quantification of Parkin W403A, F463Y, and F146A activity by MALDI-TOF auto-ubiquitylation and discharge assays

(A and B) The ligase activity of Parkin W403A was assessed using the MALDI-TOF auto-ubiquitylation assay (A) and the MALDI-TOF discharge assay (B) over a time course experiment.

(C and D) Similarly, the ligase activity of Parkin W403A, C431S, F463Y, and F146A was assessed using the MALDI-TOF autoubiquitylation assay (C) and the MALDI-TOF discharge assay (D) over a time course experiment.

(E) Detection of Parkin C431S, F463Y and F146A, and W403A autoubiquitylation species via SDS-PAGE and western blotting.

(F and G) Estimated half-maximal effective concentration of p-Ub-His for the activation of WT, W430A, and F146A Parkin using the MALDI-TOF autoubiquitylation assay (F) and the MALDI-TOF discharge assay (G). Data points are reported as the average of 3 replicates ±SD.

CellPress
OPEN ACCESS

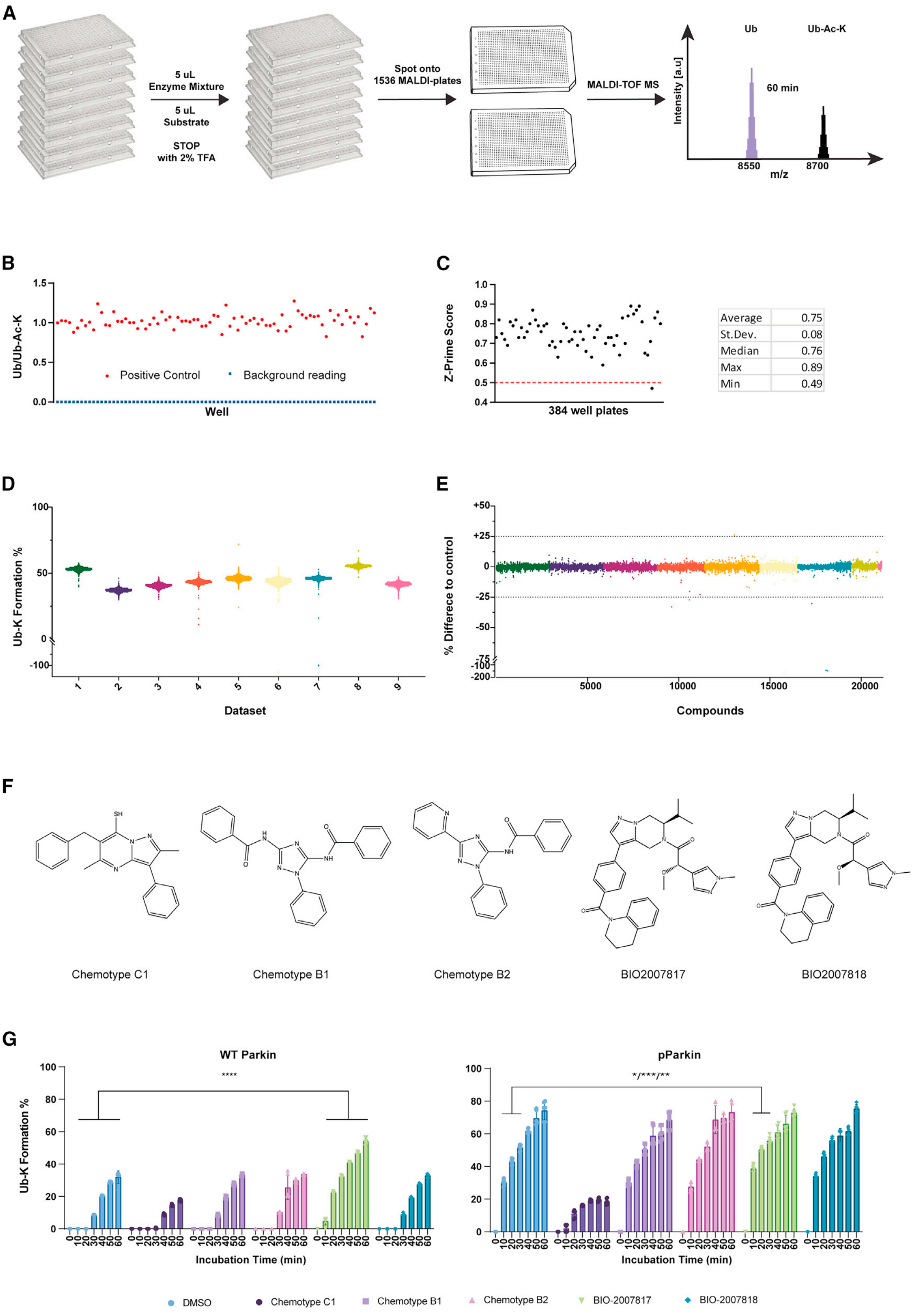

(legend on next page)

the compound-related effect. A Z' value >0.5 is considered a robust assay. The Z' average for the MALDI-TOF discharge assay was 0.75, with only one 384 plate scoring below the threshold of 0.5 (Figure 4C), confirming the robustness of the assay and the employability in HTS campaigns. A total of 60- × 384-well plates were divided into nine smaller batches of up to 8- × 384-well plates (about 2,800 compounds) to be processed daily (Figures 4D and 4E). An arbitrary and stringent hit cutoff of ±25% activity compared to the control was applied to select compounds to be further investigated. A total of 5 compounds reduced p-Parkin activity by more than 25%, and only 1 compound scored as a potential activator, for a total of 6 positive hits (Figure 4E). Two compounds were further confirmed as Parkin inhibitors by follow-up dose-response experiments (Figure S4C), indicating a true hit of 0.1%. Given the small number of compounds tested, it was not unexpected that no activating compound was detected. Identification of genuine active compounds, particularly activators, are few and far between; however, the HTS results indicated an exceptionally low false positive rate (FPR) of 0.028% and a true positive hit of 0.1%. The low false positive rate confirms the advantages of MALDI-TOF/MS-based readout compared to fluorescence-based approaches.

Validation of Parkin activators

Various Parkin activators have been mentioned in the patent literature, with only one study, conducted by Biogen and subjected to peer review, exploring the identification and optimization of Parkin activators through structure-activity relationship (SAR) analysis.[23] We examined three molecules (chemotypes B1, B2, and C1) reported in patent WO 2018/023029. Additionally, we synthesized Biogen's molecules, BIO2007818 and BIO2007817 (Figure 4F), in house to assess their ability to activate WT and p-Parkin. All compounds were tested at a final concentration of 50 μM in a time course experiment consisting of seven time points. The MALDI-TOF discharge assay was employed to measure their activation potential against WT-Parkin (activated by equimolar amounts of p-Ub-His) and p-Parkin (without p-Ub-His).

Chemotypes B1 and B2 exhibited no activating effect across more than one time point, with chemotype C1 producing instead a WT and p-Parkin inhibitory outcome (Figure 4G). BIO-2007817 produced considerably stronger Parkin activation, particularly for WT Parkin across all time points (Figure 4G, left) and for p-Parkin at time points 10, 20, and 30 min (Figure 4G, right). As previously reported, BIO-2007818 did not exhibit any p-Parkin or WT Parkin activation, aligning with its previously reported properties. Importantly, these findings further affirmed the efficacy of the MALDI-TOF discharge assay in accurately identifying Parkin activators. We conducted additional validation of BIO-2007817 results by employing SDS-PAGE followed by western blot techniques (Figures S5A and S5B) to detect the auto-ubiquitination smear of WT and p-Parkin, as well as the ubiquitylation of Miro1, a known Parkin substrate.[24,25] These experiments provided compelling evidence of a robust activation of WT Parkin and a more subtle, yet notable, activation of p-Parkin by compound BIO2007817 and closely aligned with the MALDI-TOF discharge assay results.

DISCUSSION

During the last decade, biological and structural studies have provided critical understanding of the regulation of Parkin by either direct phosphorylation on S65 or by the interaction with p-Ub. Currently, the *in vitro* quantification of Parkin's activity relies on the use of SDS-PAGE followed by antibody-based detection of ubiquitylation events. This method enables assessment of Parkin's activity via monitoring Parkin auto-ubiquitylation pattern, multi-monoubiquitylation of substrates such as MIRO1,[25] monoubiquitylation of UBE2L3,[6,8] or the formation of free Ub chains.[1,10,26] Such approaches are intrinsically low throughput and time consuming. Here, we reported two robust MALDI-TOF/MS-based assays to investigate the activity of Parkin in a fast, quantitative, and high-throughput fashion. These assays rely on quantifying Ub and Ub-K, which are easily detectable by MALDI-TOF/MS due to their small sizes, 8,565.7 Da and 8,735.7 Da, respectively. In contrast, other potential indicators of Parkin activity have considerably larger molecular weights. For instance, Parkin auto-ubiquitylation species exceed the 50 kDa molecular weight, while the multi-monoubiquitylation of substrates like MIRO1 exceeds 90 kDa, and the monoubiquitylation of UBE2L3 surpasses 20 kDa. We demonstrated that both MALDI-TOF/MS-based assays accurately and quantitatively recapitulate Parkin activity and activation rates in the presence of the activating co-factor p-Ub-His.

Parkin exists in an inactive conformation mediated by auto-inhibitory domain interfaces. Structural studies have revealed point mutations known to partially release the auto-inhibitory Parkin conformation and release some background E3 ligase activity. We employed our MALDI-TOF/MS-based strategies to enabled facile comparison and quantification of the relative impact of such point mutations on Parkin activity. In this study, we accurately quantified the impact of three Parkin-activating

Figure 4. p-Parkin HTS by MALDI-TOF discharge assay
(A) Workflow schematic.
(B) Representative data of positive controls and background reading.
(C) Z' value for HTS plates.
(D) Data distribution of independent datasets reported as violin plots. Compounds have been tested as single replicates, while 16 data points were included in each 384-well plate for both positive and negative controls.
(E) HTS data normalized to the positive controls (DMSO only) and a threshold of ±25% set for the identification of p-Parkin activators and inhibitors (dotted lines).
(F) Chemical structure of five previously reported Parkin activators.
(G) Indicated compounds were tested in a time course experiment for their effect on WT Parkin (G, left) and p-Parkin (G, right). Statistically significant activation was determined via two-way ANOVA followed by Dunnett's multiple comparisons test and indicated with asterisks for an activating effect over at least 2 consecutive time points.

CellPress
OPEN ACCESS

mutations: W403A, F146A, and F463Y. Notably, the F463Y mutant exhibited activating properties, albeit lower activity in substrate depletion and discharge, when compared to the W403A and F146A mutants. These findings are consistent with previous literature on the subject. Interestingly, the disparity in p-Ub-His EC50 appears to be primarily attributed to the auto-ubiquitylation step rather than the discharge on free lysine step. Both the W403A and F146A mutants display a similar p-Ub-His dependency for discharge, suggesting that these specific mutations affect the conformational changes of Parkin involved in self-charging.

While this assay will aid in understanding the regulation of Parkin activity by academic researchers, MALDI-TOF/MS-based technologies are emerging as the gold standard in the drug discovery space. Fluorescence probes such as UbMes and UbFluor have been reported as functional Ub-based probes for determining Parkin activity.[11] Such strategies are potentially scalable to HTS levels; however, the use of fluorescence as analytical readout is inherently problematic because of fluorescence artifacts that result in both false positives and false negatives. For example, UbFluor is labile in the presence of reducing agents or other small molecules that possess thiol or amine groups that may cleave UbFluor even in the absence of RBR E3, resulting in false positives.[10] Fluorescent small molecules may also disrupt fluorescence polarization readings, resulting in false negatives. The rates of false positives and negatives are highly dependent on the fluorophore used, the stability of the substrate, the assay conditions, and the nature of the chemical libraries tested. A recent study suggested that the false discovery rate might score anywhere between 0.5% and 9.9% depending on the assay and type of fluorescence used.[27] This translates into the risk of following up on false leads, with obvious consequences in terms of increased costs and reduced efficiency. The screening of ~20,000 compounds using the MALDI-TOF discharge assay resulted in a false positive rate of only 0.028%, well below what is expected with fluorescent-based tools.

The deficiency of Parkin function plays a significant role in the development of PD. To combat PD effectively, there is considerable promise in developing pharmaceutical compounds that can restore normal levels of mitophagy. The social and economic impact of PD has spurred intense research efforts to identify pharmacological treatments, leading to several patents that report chemical structures of Parkin activators.

In our study, we investigated five previously reported Parkin-activating small molecules using the MALDI-TOF discharge assay. Among these compounds, three were from patent literature (chemotypes B1, B2, and C1), and the other two were recently peer-reviewed structures that underwent SAR optimization (BIO-2007817 and BIO-2007818). Utilizing the MALDI-TOF discharge assay, we validated the anticipated activation effect of BIO-2007817 on both WT Parkin and p-Parkin. Notably, BIO-2007817 demonstrated robust activation of WT Parkin and displayed some activity on p-Parkin as well.

Importantly, the HTS MALDI-TOF/MS-based strategy presented in this study can be readily applied to other RBR E3 ligases, including those E3 ligases that discharge on non-canonical residues (e.g., serine and sugars), such as HOIL-1,[28–30] RNF213,[31] and RNF216.[32] Overall, we anticipate that MALDI-TOF/MS-based technologies will significantly enhance our understanding of the functioning of E2-conjugating enzymes and E3 ligases by providing accurate and quantitative data. Moreover, these techniques are poised to contribute significantly to drug discovery campaigns in the Ub field.

Limitations of the study

In the current study, we reported the conceptualization, validation, and high-throughput scale up of two MALDI-TOF/MS-based assays for testing Parkin activity and the identification of Parkin activators: the MALDI-TOF auto-ubiquitylation and discharge assay. We identified the MALDI-TOF discharge assay as better suited for the identification of Parkin activators in a high-throughput setting compared to the auto-ubiquitination assay.

A constraint of our HTS approach is the utilization of approximately 70% p-Parkin. Consequently, potential Parkin activators identified through this method may necessitate additional deconvolution assays to determine whether the activation is mediated by p-Parkin or its native counterpart. Addressing this can be accomplished through subsequent testing of high-throughput screening-positive hits against both WT Parkin and fully phosphorylated Parkin.

The impact of BIO2007817 was noticeable on both WT and p-Parkin. However, its effect on the fully activated p-Parkin resulted in a modest increase in activity, ranging between 10% and 25%, only within the initial three time points of the time course experiment (Figure 4G; Figure S5B). These findings suggest a potential for improving the assay's sensitivity and broadening its dynamic range. The availability of highly optimized Parkin activators offers an opportunity to refine experimental parameters, including optimizing incubation time, temperature, and enzymatic and substrate concentrations to enhance the assay's sensitivity and widening its dynamic range. Additionally, it is important to note a limitation regarding the relatively small size of the tested compound library. To address this, expanding both the library size and the diversity within the chemical space, in conjunction with the application of experimentally optimized conditions specifically tailored for detecting WT and p-Parkin activation, will significantly enhance our ability to discover new Parkin activators.

STAR★METHODS

Detailed methods are provided in the online version of this paper and include the following:

- KEY RESOURCES TABLE
- RESOURCE AVAILABILITY
  - Lead contact
  - Materials availability
  - Data and code availability
- EXPERIMENTAL MODEL AND STUDY PARTICIPANT DETAILS
  - Bacterial models

- METHOD DETAILS
  - Autoubiquitylation MALDI-TOF/MS Parkin activity assay
  - Discharge MALDI-TOF/MS Parkin activity assay
  - Parkin HTS screening
  - Expression and purification of recombinant GST-PINK1 126-end (Pediculus humanus)
  - Expression and purification of recombinant Parkin 1-465 (human), Parkin active mutants and p-Parkin
  - Expression and purification of recombinant p-Ubiquitin-His (pSer65-ubiquitin-6His), ^{15}N Ubiquitin and Ub-K
  - Parkin Ubiquitylation assay
- QUANTIFICATION AND STATISTICAL ANALYSIS

SUPPLEMENTAL INFORMATION

Supplemental information can be found online at https://doi.org/10.1016/j.crmeth.2024.100712.

ACKNOWLEDGMENTS

We thank the MRC-PPU Mass Spectrometry facility (Dr. Renata Soares, Robert Gourlay, and Joby Varghese), the former and current MRC-PPU Head of Chemistry, Dr. Natalia Shpiro and Dr. Robin Pflughaupt, and the MRC-PPU Reagents and Service team, particularly Clare Johnson, for their technical support. This work was supported by a grant from The Michael J. Fox Foundation for Parkinson's Research to Vincere Biosciences. V.D.C. is supported by UKRI Grant Reference MR/V025759/1, M.M.K.M. is supported by a Wellcome Trust Senior Research Fellowship in Clinical Science (grant reference 210753/18/2), The Michael J. Fox Foundation, and an EMBO YIP Award. We acknowledge funding from companies supporting the Division of Signal Transduction Therapy (Boehringer-Ingelheim, Glaxo Smith Kline, and Merck KGaA). The graphical abstract was created with BioRender.com.

AUTHOR CONTRIBUTIONS

Conceptualization, K.M., M.M.K.M., and V.D.C.; methodology, R.T., J.M., and V.D.C.; validation, M.S. and O.A.; formal analysis, R.T., J.M., O.A., and V.D.C.; investigation, R.T., J.M., M.S., O.A., A.K., and V.D.C.; resources, M.S., O.A., and A.K.; data curation, R.T., O.A., and V.D.C.; writing – original draft, M.M.K.M., B.B., K.M., and V.D.C.; writing – review & editing, M.M.K.M., B.B., K.M., and V.D.C.; visualization, R.T., O.A., and V.D.C.; supervision, B.B., K.M., C.J.H., P.D., M.M.K.M., and V.D.C.; project administration, B.B., K.M., C.J.H, P.D., M.M.K.M., and V.D.C.; funding acquisition, B.B., K.M., C.J.H, P.D., M.M.K.M., and V.D.C.

DECLARATION OF INTERESTS

B.B. is a shareholder and CEO of Vincere Biosciences, Inc., focused on developing therapeutics targeting mitochondrial pathways. When this study was initiated, K.M. was the CSO at Vincere, accountable for drug discovery. K.M. is an advisor to The Michael J. Fox Foundation, serves on advisory panels at the National Institutes of Health, and consults for several biopharmaceutical companies. M.M.K.M. is a member of the scientific advisory board of Montara Therapeutics, Inc and scientific consultant at MSD. R.T. is an employee of the DUB Profiling Service within the MRC-PPU Reagents and Services (https://mrcppureagents.dundee.ac.uk/). C.J.H. is manager of the MRC-PPU Reagents and Services.

Received: February 23, 2022
Revised: October 11, 2023
Accepted: January 26, 2024
Published: February 20, 2024

REFERENCES

1. Narendra, D.P., and Youle, R.J. (2011). Targeting mitochondrial dysfunction: role for PINK1 and Parkin in mitochondrial quality control. Antioxidants Redox Signal. *14*, 1929–1938. https://doi.org/10.1089/ars.2010.3799.
2. Koyano, F., Okatsu, K., Kosako, H., Tamura, Y., Go, E., Kimura, M., Kimura, Y., Tsuchiya, H., Yoshihara, H., Hirokawa, T., et al. (2014). Ubiquitin is phosphorylated by PINK1 to activate parkin. Nature *510*, 162–166. https://doi.org/10.1038/nature13392.
3. Shiba-Fukushima, K., Imai, Y., Yoshida, S., Ishihama, Y., Kanao, T., Sato, S., and Hattori, N. (2012). PINK1-mediated phosphorylation of the Parkin ubiquitin-like domain primes mitochondrial translocation of Parkin and regulates mitophagy. Sci. Rep. *2*, 1002. https://doi.org/10.1038/srep01002.
4. Chaugule, V.K., Burchell, L., Barber, K.R., Sidhu, A., Leslie, S.J., Shaw, G.S., and Walden, H. (2011). Autoregulation of Parkin activity through its ubiquitin-like domain. EMBO J. *30*, 2853–2867. https://doi.org/10.1038/emboj.2011.204.
5. Kumar, A., Aguirre, J.D., Condos, T.E.C., Martinez-Torres, R.J., Chaugule, V.K., Toth, R., Sundaramoorthy, R., Mercier, P., Knebel, A., Spratt, D.E., et al. (2015). Disruption of the autoinhibited state primes the E3 ligase parkin for activation and catalysis. EMBO J. *34*, 2506–2521. https://doi.org/10.15252/embj.201592337.
6. Trempe, J.F., Sauvé, V., Grenier, K., Seirafi, M., Tang, M.Y., Ménade, M., Al-Abdul-Wahid, S., Krett, J., Wong, K., Kozlov, G., et al. (2013). Structure of parkin reveals mechanisms for ubiquitin ligase activation. Science *340*, 1451–1455. https://doi.org/10.1126/science.1237908.
7. Sauvé, V., Lilov, A., Seirafi, M., Vranas, M., Rasool, S., Kozlov, G., Sprules, T., Wang, J., Trempe, J.F., and Gehring, K. (2015). A Ubl/ubiquitin switch in the activation of Parkin. EMBO J. *34*, 2492–2505. https://doi.org/10.15252/embj.201592237.
8. Kondapalli, C., Kazlauskaite, A., Zhang, N., Woodroof, H.I., Campbell, D.G., Gourlay, R., Burchell, L., Walden, H., Macartney, T.J., Deak, M., et al. (2012). PINK1 is activated by mitochondrial membrane potential depolarization and stimulates Parkin E3 ligase activity by phosphorylating Serine 65. Open Biol. *2*, 120080. https://doi.org/10.1098/rsob.120080.
9. Kazlauskaite, A., Kondapalli, C., Gourlay, R., Campbell, D.G., Ritorto, M.S., Hofmann, K., Alessi, D.R., Knebel, A., Trost, M., and Muqit, M.M.K. (2014). Parkin is activated by PINK1-dependent phosphorylation of ubiquitin at Ser65. Biochem. J. *460*, 127–139. https://doi.org/10.1042/BJ20140334.
10. Foote, P.K., and Statsyuk, A.V. (2018). Monitoring PARKIN RBR Ubiquitin Ligase Activation States with UbFluor. Curr. Protoc. Chem. Biol. *10*, e45. https://doi.org/10.1002/cpch.45.
11. Park, S., Foote, P.K., Krist, D.T., Rice, S.E., and Statsyuk, A.V. (2017). UbMES and UbFluor: Novel probes for ring-between-ring (RBR) E3 ubiquitin ligase PARKIN. J. Biol. Chem. *292*, 16539–16553. https://doi.org/10.1074/jbc.M116.773200.
12. De Cesare, V., Johnson, C., Barlow, V., Hastie, J., Knebel, A., and Trost, M. (2018). The MALDI-TOF E2/E3 Ligase Assay as Universal Tool for Drug Discovery in the Ubiquitin Pathway. Cell Chem. Biol. *25*, 1117–1127.e4. https://doi.org/10.1016/j.chembiol.2018.06.004.
13. De Cesare, V. (2023). MALDI-TOF Mass Spectrometry for interrogating ubiquitin enzymes. Front Mol Biosci *10*, 1184934. https://doi.org/10.3389/fmolb.2023.1184934.
14. Wenzel, D.M., Lissounov, A., Brzovic, P.S., and Klevit, R.E. (2011). UBCH7 reactivity profile reveals parkin and HHARI to be RING/HECT hybrids. Nature *474*, 105–108. https://doi.org/10.1038/nature09966.
15. Kazlauskaite, A., Martínez-Torres, R.J., Wilkie, S., Kumar, A., Peltier, J., Gonzalez, A., Johnson, C., Zhang, J., Hope, A.G., Peggie, M., et al. (2015). Binding to serine 65-phosphorylated ubiquitin primes Parkin for optimal PINK1-dependent phosphorylation and activation. EMBO Rep. *16*, 939–954. https://doi.org/10.15252/embr.201540352.

CellPress
OPEN ACCESS

16. Wauer, T., and Komander, D. (2013). Structure of the human Parkin ligase domain in an autoinhibited state. EMBO J. *32*, 2099–2112. https://doi.org/10.1038/emboj.2013.125.

17. Riley, B.E., Lougheed, J.C., Callaway, K., Velasquez, M., Brecht, E., Nguyen, L., Shaler, T., Walker, D., Yang, Y., Regnstrom, K., et al. (2013). Structure and function of Parkin E3 ubiquitin ligase reveals aspects of RING and HECT ligases. Nat. Commun. *4*, 1982. https://doi.org/10.1038/ncomms2982.

18. Tang, M.Y., Vranas, M., Krahn, A.I., Pundlik, S., Trempe, J.F., and Fon, E.A. (2017). Structure-guided mutagenesis reveals a hierarchical mechanism of Parkin activation. Nat. Commun. *8*, 14697. https://doi.org/10.1038/ncomms14697.

19. Stevens, M.U., Croteau, N., Eldeeb, M.A., Antico, O., Zeng, Z.W., Toth, R., Durcan, T.M., Springer, W., Fon, E.A., Muqit, M.M., and Trempe, J.F. (2023). Structure-based design and characterization of Parkin-activating mutations. Life Sci. Alliance *6*, e202201419. https://doi.org/10.26508/lsa.202201419.

20. Kitada, T., Asakawa, S., Hattori, N., Matsumine, H., Yamamura, Y., Minoshima, S., Yokochi, M., Mizuno, Y., and Shimizu, N. (1998). Mutations in the parkin gene cause autosomal recessive juvenile parkinsonism. Nature *392*, 605–608. https://doi.org/10.1038/33416.

21. Valente, E.M., Abou-Sleiman, P.M., Caputo, V., Muqit, M.M.K., Harvey, K., Gispert, S., Ali, Z., Del Turco, D., Bentivoglio, A.R., Healy, D.G., et al. (2004). Hereditary early-onset Parkinson's disease caused by mutations in PINK1. Science *304*, 1158–1160. https://doi.org/10.1126/science.1096284.

22. Miller, S., and Muqit, M.M.K. (2019). Therapeutic approaches to enhance PINK1/Parkin mediated mitophagy for the treatment of Parkinson's disease. Neurosci. Lett. *705*, 7–13. https://doi.org/10.1016/j.neulet.2019.04.029.

23. Shlevkov, E., Murugan, P., Montagna, D., Stefan, E., Hadzipasic, A., Harvey, J.S., Kumar, P.R., Entova, S., Bansal, N., Bickford, S., et al. (2022). Discovery of small-molecule positive allosteric modulators of Parkin E3 ligase. iScience *25*, 103650. https://doi.org/10.1016/j.isci.2021.103650.

24. Sarraf, S.A., Raman, M., Guarani-Pereira, V., Sowa, M.E., Huttlin, E.L., Gygi, S.P., and Harper, J.W. (2013). Landscape of the PARKIN-dependent ubiquitylome in response to mitochondrial depolarization. Nature *496*, 372–376. https://doi.org/10.1038/nature12043.

25. Kazlauskaite, A., Kelly, V., Johnson, C., Baillie, C., Hastie, C.J., Peggie, M., Macartney, T., Woodroof, H.I., Alessi, D.R., Pedrioli, P.G.A., and Muqit, M.M.K. (2014). Phosphorylation of Parkin at Serine65 is essential for activation: elaboration of a Miro1 substrate-based assay of Parkin E3 ligase activity. Open Biol. *4*, 130213. https://doi.org/10.1098/rsob.130213.

26. Antico, O., Ordureau, A., Stevens, M., Singh, F., Nirujogi, R.S., Gierlinski, M., Barini, E., Rickwood, M.L., Prescott, A., Toth, R., et al. (2021). Global ubiquitylation analysis of mitochondria in primary neurons identifies endogenous Parkin targets following activation of PINK1. Sci. Adv. *7*, eabj0722. https://doi.org/10.1126/sciadv.abj0722.

27. Borrel, A., Huang, R., Sakamuru, S., Xia, M., Simeonov, A., Mansouri, K., Houck, K.A., Judson, R.S., and Kleinstreuer, N.C. (2020). High-Throughput Screening to Predict Chemical-Assay Interference. Sci. Rep. *10*, 3986. https://doi.org/10.1038/s41598-020-60747-3.

28. Kelsall, I.R., Zhang, J., Knebel, A., Arthur, J.S.C., and Cohen, P. (2019). The E3 ligase HOIL-1 catalyses ester bond formation between ubiquitin and components of the Myddosome in mammalian cells. Proc. Natl. Acad. Sci. USA *116*, 13293–13298. https://doi.org/10.1073/pnas.1905873116.

29. Petrova, T., Zhang, J., Nanda, S.K., Figueras-Vadillo, C., and Cohen, P. (2021). HOIL-1-catalysed, ester-linked ubiquitylation restricts IL-18 signaling in cytotoxic T cells but promotes TLR signalling in macrophages. FEBS J. *288*, 5909–5924. https://doi.org/10.1111/febs.15896.

30. Kelsall, I.R., McCrory, E.H., Xu, Y., Scudamore, C.L., Nanda, S.K., Mancebo-Gamella, P., Wood, N.T., Knebel, A., Matthews, S.J., and Cohen, P. (2022). HOIL-1 ubiquitin ligase activity targets unbranched glucosaccharides and is required to prevent polyglucosan accumulation. EMBO J. *41*, e109700. https://doi.org/10.15252/embj.2021109700.

31. Otten, E.G., Werner, E., Crespillo-Casado, A., Boyle, K.B., Dharamdasani, V., Pathe, C., Santhanam, B., and Randow, F. (2021). Ubiquitylation of lipopolysaccharide by RNF213 during bacterial infection. Nature *594*, 111–116. https://doi.org/10.1038/s41586-021-03566-4.

32. Wang, X.S., Cotton, T.R., Trevelyan, S.J., Richardson, L.W., Lee, W.T., Silke, J., and Lechtenberg, B.C. (2023). The unifying catalytic mechanism of the RING-between-RING E3 ubiquitin ligase family. Nat. Commun. *14*, 168. https://doi.org/10.1038/s41467-023-35871-z.

33. Ritorto, M.S., Ewan, R., Perez-Oliva, A.B., Knebel, A., Buhrlage, S.J., Wightman, M., Kelly, S.M., Wood, N.T., Virdee, S., Gray, N.S., et al. (2014). Screening of DUB activity and specificity by MALDI-TOF mass spectrometry. Nat. Commun. *5*, 4763. https://doi.org/10.1038/ncomms5763.

34. De Cesare, V., Carbajo Lopez, D., Mabbitt, P.D., Fletcher, A.J., Soetens, M., Antico, O., Wood, N.T., and Virdee, S. (2021). Deubiquitinating enzyme amino acid profiling reveals a class of ubiquitin esterases. Proc. Natl. Acad. Sci. USA *118*, e2006947118. https://doi.org/10.1073/pnas.2006947118.

STAR★METHODS

KEY RESOURCES TABLE

REAGENT or RESOURCE	SOURCE	IDENTIFIER
**Antibodies**		
Mouse Anti-6X His tag Clone HIS.H8	Abcam	Cat#ab18184<br>RRID:AB_444306
anti-Ubiquitin	BioLegend	Cat#646302<br>RRID:AB_1659269
RHOT1 monoclonal antibody (M01), clone 4H4	Abnova	Cat# H00055288-M01<br>RRID:AB_606929
Parkin (Prk8) Mouse monoclonal Antibody	Cell signaling technologies	Cat#4211<br>RRID:AB_2159920
Donkey Anti-Mouse Alexa Fluor 488	Thermo Fisher Scientific	Cat#A32766<br>RRID:AB_2762823
**Bacterial and virus strains**		
E. coli BL21 codon plus cells.	New England Biolabs	Cat# C2527H
**Chemicals, peptides, and recombinant proteins**		
Ubiquitin (including pSer65 and 15N labeled)	MRC-PPU reagents	DU20027
Ubiquitin-His6 (pSer65)	MRC-PPU reagents	DU21990
His-UBE1	MRC-PPU reagents	DU32888
UBE2L3	MRC PPU reagents	DU3772
Parkin WT	MRC-PPU reagents	DU40847
Parkin WT (pSer 65)	MRC-PPU reagents	DU40847
Parkin [F146A]	MRC-PPU reagents	DU44642
Parkin [W403A]	MRC-PPU reagents	DU44643
Parkin [F463Y]	MRC-PPU reagents	DU58844
Parkin [C431S]	MRC-PPU reagents	DU39784
GST-PINK1	MRC-PPU reagents	DU34798
Miro1	MRC PPU reagents	DU43034
Ac-K	Bachem/Cambridge	Cat. Number 4000486.0001
**Deposited data**		
Raw and analyzed data	This paper	https://doi.org/10.5281/zenodo.10402851
**Recombinant DNA**		
pET15b His-SUMO-Parkin WT	MRC-PPU reagents	DU40847
pET15b His-SUMO-Parkin WT (pSer 65)	MRC-PPU reagents	DU40847
pET15b-His-SUMO Parkin [F146A]	MRC-PPU reagents	DU44642
pET15b-His-SUMO-Parkin [W403A]	MRC-PPU reagents	DU44643
pET15b His-SUMO-Parkin [F463Y]	MRC-PPU reagents	DU58844
pET15b His-Sumo-Parkin [C431S]	MRC-PPU reagents	DU39796
FastBac HTb His-UBE1	MRC-PPU reagents	DU32888
pET24a-Ubiquitin-SATGSHHHHHHG	MRC-PPU reagents	DU21990
pET28a FLAG-GG-Ubiquitin	MRC-PPU reagent	DU46789
pET24 Ubiquitin (1–76)	MRC-PPU reagent	DU20027
pGEX6P-PINK1 (p. h)	MRC-PPU reagents	DU34798
pET156P UBE2L3	MRC-PPU reagent	DU12798
**Software and algorithms**		
GraphPad Prism version 10.0.3	https://www.graphpad.com/	N/A
Excel Office	Microsoft, WA, USA	https://www.microsoft.com/
Adobe Illustrator 2021	Adobe Systems, CA, USA	https://www.adobe.com/products/illustrator

(*Continued on next page*)

CellPress
OPEN ACCESS

Continued

REAGENT or RESOURCE	SOURCE	IDENTIFIER
FlexControl Version 4.2 (Build 81)	Bruker Daltonik GmbH	N/A
FlexAnalysis Version 4.2 (Build 14)	Bruker Daltonik GmbH	N/A

RESOURCE AVAILABILITY

Lead contact

Further information and requests for resources and reagents should be directed to and will be fulfilled by the lead contact, Virginia De Cesare (vdecesare@dundee.ac.uk).

Materials availability

All plasmids generated in this study are available to MRC-PPU Reagents and Services (https://mrcppureagents.dundee.ac.uk/). Unique Identifiers are reported in the key resources table.

Data and code availability

- Original dataset and raw data of western blot acquisitions have been deposited at Zenodo and are publicly available as of the date of publication. DOIs are listed in the key resources table.
- This paper does not report original code.
- Any additional information required to reanalyse the data reported in this paper is available from the lead contact upon request.

EXPERIMENTAL MODEL AND STUDY PARTICIPANT DETAILS

Bacterial models

Details regarding the E. coli strains utilized in this study and the plasmids employed for transfection and protein expression are outlined in the key resources table. Transfection of *E. coli* BL21 codon plus cells adhered to standard protocols. Briefly, *E. coli* BL21 codon plus cells were thawed on ice. Subsequently, 1–5 μL containing 1 pg–100 ng of plasmid DNA was gently mixed with the *E. coli* BL21 codon plus cells and left on ice for 30 min. The mixture underwent a brief exposure to 42°C for 10 s, followed by another period on ice for 5 min. Following this, 950 μL of SOC media was introduced to the mixture, which was then incubated at 37°C for 60 min. 50–100 μL of the mixture was spread onto a selection plate and left to incubate overnight at 37°C. A single antibiotic-resistant colony was selected and propagated for 16 h at 37°C under agitation at 200 rpm. The cells underwent successive propagation, and protein expression was induced using plasmid-specific procedures, as described in the methods details section.

METHOD DETAILS

Autoubiquitylation MALDI-TOF/MS Parkin activity assay

200 nM UBE1 activating enzyme, 1000 nM UBE2L3 conjugating enzyme, 1000 nM WT parkin or p-Parkin, 20 mM $MgCl_2$, 2 mM ATP, 0.05% BSA and 2 mM TCEP were mixed in 1X phosphate buffer (PBS, pH 8.5) and aliquoted into Eppendorf Low-Bind plates (5 μL per well). The reactions were started by adding 5 μL of 50 μM Ubiquitin (in 1X PBS, pH 8.5) supplemented with the indicated amount of p-Ub-His. Plates were sealed with adhesive aluminum foil and incubated at 37°C in an Eppendorf ThermoMixer C (Eppendorf) equipped with a ThermoTop and a SmartBlock PCR 384. The reactions were stopped at the indicated time points by the addition of 5 μL 6% TFA supplemented with 6 μM ^{15}N Ubiquitin. Samples were spotted on 1536 AnchorChip MALDI target using a Mosquito nanoliter pipetting system (TTP Labtech) and analyzed by MALDI-TOF/MS as previously reported.[12] Briefly, samples were analyzed using a Rapiflex MALDI-TOF/MS equipped with Compass for flexSeries 2.0 (flexControl Version 4.0 – Build 48) using automated runs. The automated method was set in positive mode, detection window between 8.2 and 9 kDa, Sample Rate and Digitizer Setting 5.00 GS/s. Movement on Sample Spot set as Random – Complete sample Mode with 4000 shots at raster spot and diameter limit to 800 μm. Peak Detection in centroid mode and a 5.0 Signal to Noise threshold. Spectra automated analysis was performed using FlexAnalysis (Version 4.0, Build 14). The processing method used Snap as Peal Detection Algorithm, Averagine as SNAP average composition and baseline Substraction set on TopHat. SavitzkyGolay was used as smoothing algorithm using width 0.2 m/z and Cycles value set on 1. Observed molecular weight of Ubiquitin (8565.76 Da), ^{15}N Ubiquitin (8669.47 Da) and Ub-K (8735.74 Da) were used in the Mass Control List as internal calibrant, depending on the type of experiment (either auto-ubiquitylation of ubiquitin discharge on Ac-lysine).

Discharge MALDI-TOF/MS Parkin activity assay

An identical enzymatic mixture as the autoubiquitylation assay was prepared. The reactions were started by adding 5 μL of 50 μM Ubiquitin supplemented with the indicated amount of p-Ub-His and 50 mM Ac-K. Plates were incubated at room temperature (25°) and sealed with adhesive aluminum foil. The reactions were stopped at the indicated time points by the addition of 5 μL 6% TFA.

Parkin HTS screening

All Parkin HTS assays were performed in a total volume of 20.01 μL at room temperature using a FluidX Xrd-384 dispenser. To plates containing 20 nL of compound 10 μl of a mix containing 500 nM p-PARKIN, 400 nM UBE1, 4000 nM UBE2L3, 20 μM MgCl2, 2 mM ATP in a 50 mM HEPES pH 8.5 20 mM TECEP buffer was added. The plates were preincubated at 25°C for 30mins and the assay was then initiated with the addition of 10 μL of Ubiquitin mix containing 100 μM Ubiquitin, 100 mM Ac-lysine. The assay was incubated for 20 min at 25°C. The assay was then terminated with the addition of 10 μL 6% TFA.

Expression and purification of recombinant GST-PINK1 126-end (Pediculus humanus)

E. coli BL21 codon plus cells were transformed with MRC-PPU plasmid DU34798. A single antibiotic resistant colony was selected and propagated for 16 h at 37°C, 200 rpm. 12 x 1L batches of LB broth/carbenicillin were inoculated with the overnight culture and grown until an OD_{600} of 0.8. The incubation temperature was dropped to 26°C and PINK1 expression was induced by supplementing the media with 0.1 mM Isopropyl β-D-1-thiogalactopyranoside (IPTG) and left to express for overnight. The cells were collected by centrifugation (25 min at 4200 rpm) and the clarified broth was decanted. The cells were resuspended in 20 mL per pellet of 50 mM Tris pH 7.5, 250 mM NaCl, 1 mM DTT, 1 mM AEBSF, 10 μg/mL Leupeptin. The suspension was collected into 50 mL centrifuge vials, chilled on ice and sonicated using 6 pulses of 55% amplitude and 15 s pulses. The suspension was clarified by centrifugation at 40000 x g for 25 min at 4°C. 6 mL GSH-agarose was equilibrated with wash buffer (50 mM Tris pH 7.5, 250 mM NaCl, 1 mM DTT) and mixed with the clarified cell lysate for 90 min. The GSH-agarose was recovered by sedimentation, washed 5 times with 5 volumes of wash buffer and eluted in wash buffer containing 10 mM reduced GSH.

Expression and purification of recombinant Parkin 1-465 (human), Parkin active mutants and p-Parkin

Human wild type Parkin 1-465 along with the F146A, W403A, and F463Y mutants (MRC-PPU plasmids DU40847, DU44642, DU44643 and DU58844) were expressed as His6-SUMO-fusion proteins and purified as described previously[8] using *E. coli* BL21 codon plus cells.

To produce phosphorylated Parkin, the fusion protein was captured on Ni-agarose, washed, and incubated with 5 mg of GST-PINK1 126-end in the presence of 10 mM $MgCl_2$ and 2 mM ATP for 4 h at 27°C. The initial kinase and Mg-ATP were removed and replaced with fresh kinase and Mg-ATP for incubation over night at 27°C. The Ni-agarose was washed three times with wash buffer and Parkin was eluted in the smallest possible volume. The protein was then dialyzed in the presence of SENP1 as previously described[8,9] The protein was further phosphorylated with more PINK1 and Mg-ATP and at the same time concentrated to 6 mg/mL. Finally, the protein was purified further by chromatography on a Superdex 200 as described above and concentrated to about 2 mg/mL.

Expression and purification of recombinant p-Ubiquitin-His (pSer65-ubiquitin-6His), ^{15}N Ubiquitin and Ub-K

Ubiquitin-His_6 was produced from a kanamycin resistance conferring plasmid MRC-PPU reagent DU21990 using *E. coli* BL21 codon plus cells. The cells were grown in autoinduction media supplemented with 50 μg/mL kanamycin to an OD600 of 1.0 and were further allowed to shake overnight at 16°C. The cells were harvested the following morning and lysed in 50 mM Tris pH 7.5, 250 mM NaCl, 25 mM imidazole, 7 mM 2-mercaptoethanol, 10 μg/mL Leupeptin (Apollo Scientific), 1 mM AEBSF (Apollo Scientific). The protein was purified over Ni-NTA agarose, eluted into a 0.4 M imidazole buffer and dialyzed against 50 mM Tris pH 7.5, 200 mM Tris pH 7.5, 7 mM 2-mercapto ethanol. For phosphorylation at Ser65, 20 mg of Ubiquitin-His was incubated with 2 mg of GST-PINK1 in the presence of 10 mM $MgCl_2$ and 2 mM ATP for overnight at 28°C. The Ubiquitin-His was collected on 1 mL Ni-NTA agarose, washed 4 times with 12 bed volumes of 50 mM Tris pH 7.5, 200 mM Tris pH 7.5, 7 mM 2-mercapto ethanol and recovered by elution with imidazole. Imidazole was removed and p-Ub-His concentrated using Millipore Ultra filter (3000 MWCO) followed by subsequent sample dilution in 1x PBS, pH 7.0. The sequence was repeat for 6 times using a 6-fold dilution. Phosphorylation efficiency of both p-Ub-His and p-Ub were assessed by LC-MS analysis (Sup. Figure 1): 5 μL of a 0.2 μg/μL solution were injected into an LC-MS Agilent Technologies 1260 Infinity Liquid chromatography System equipped with Zorbax 300SB-C3 5uM 2.1 × 150 mm column and a 6130 Single Quadrupole. Samples were run using an Acetonitrile gradient from 10 to 75% over 20 min. Spectra were automatically deconvoluted using Software ChemStation Rev. B. 04. 03-SP1 and the following settings were used to evaluate the spectra: MW Agreement set to 0.05%, Noise Cutoff to 1000 counts, Abundance Cutoff to 10%. The estimated phosphorylation of p-Ub-His (9812.24 Da) occurred at approximately 70%. This estimation was made based on the relative abundance of unphosphorylated ubiquitin (9732.14 Da), which was found to constitute 33.7% of the main peak. No further purification step was performed, the relative purity was considered in the experimental calculations. Expression and purification of ^{15}N-Ubiquitin and Ub-K was performed as previously reported.[33,34]

Parkin Ubiquitylation assay

In vitro ubiquitylation assays were performed using recombinant proteins purified from *E. coli* BL21 codon plus cells unless stated otherwise. In a final volume of 50 μL, an ubiquitin master mix [50 mM Tris-HCl pH 7.5, 5 mM MgCl2, 2 mM ATP, 0.1 μM His-UBE1 expressed in Sf21 insect cells, 1 μM human UBE2L3, 50 μM Flag-ubiquitin) was used with 62.5 nM of wild type or pS65 Parkin and incubated with 0.5 μM Miro recombinant protein, either with or without 62 nM of pUbiquitin at 37°C for 30 min in a thermoshaker at 1100 rpm. Kinetic ubiquitylation profile of BIO-2007817 (100 μM) was evaluated at 0, 10, 20, 30 and 60 min. DMSO was used as vehicle. Reactions were terminated by the addition of 4 x LDS loading buffer plus β-mercaptoethanol. 1/3 volume of the reactions

were resolved using SDS-PAGE on 4–12% Bis-Tris gels in MOPS buffer and transferred to nitrocellulose membranes. Membranes were blocked with 5% milk powder in TBS +0.1% TWEEN 20 (TBS-T) for 1h at room temperature then immunoblotted against the primary antibody in 5% BSA/TBS-T at 4°C overnight. Protein bands were detected by blotting against secondary antibodies labeled with 800 nm or 680 nm fluorophores in TBS-T for 1h at room temperature and imaged using LiCor. Primary antibodies anti-His tag (Abcam ab18184), anti-Ubiquitin (BioLegend 646302), anti-Miro (Abnova H00055288-M01) antibodies, anti-Parkin (Prk8-Cell Signaling Technology 4211).

QUANTIFICATION AND STATISTICAL ANALYSIS

Data obtained from the MALDI-TOF autoubiquitylation assay underwent normalization utilizing the ^{15}N ubiquitin signal, employing the formula:

$$\frac{\frac{Ubi}{15N\ Ubi}\ sample}{\frac{Ubi}{15N\ Ubi}\ control}\ x\ 100$$

For data derived from the MALDI-TOF discharge assay, the conversion to % of Ub-K formation was achieved using the formula outlined in Figure S4B.

The Z′ Prime Score was computed through the standard formula:

$$Z' = 1 - \left(\frac{3(\sigma positive + \sigma negative)}{|\mu positive - \mu negative|}\right)$$

Where σ positive denotes the standard deviation of the positive control, σ negative signifies the standard deviation of the negative control, μ positive represents the mean of the positive control μ negative stands for the mean of the negative control.

Statistically significant activation of WT and p-Parkin mediated by small molecules was determined via two-way ANOVA followed by Dunnett's multiple comparisons test and indicated with asterisks for activating effect over at least 2 consecutive time points.

Cell Reports Methods, Volume *4*

Supplemental information

Design and high-throughput implementation of MALDI-TOF/MS-based assays for Parkin E3 ligase activity

Ryan Traynor, Jennifer Moran, Michael Stevens, Odetta Antico, Axel Knebel, Bahareh Behrouz, Kalpana Merchant, C. James Hastie, Paul Davies, Miratul M.K. Muqit, and Virginia De Cesare

Supplementary Figures

Sup. Figure 1

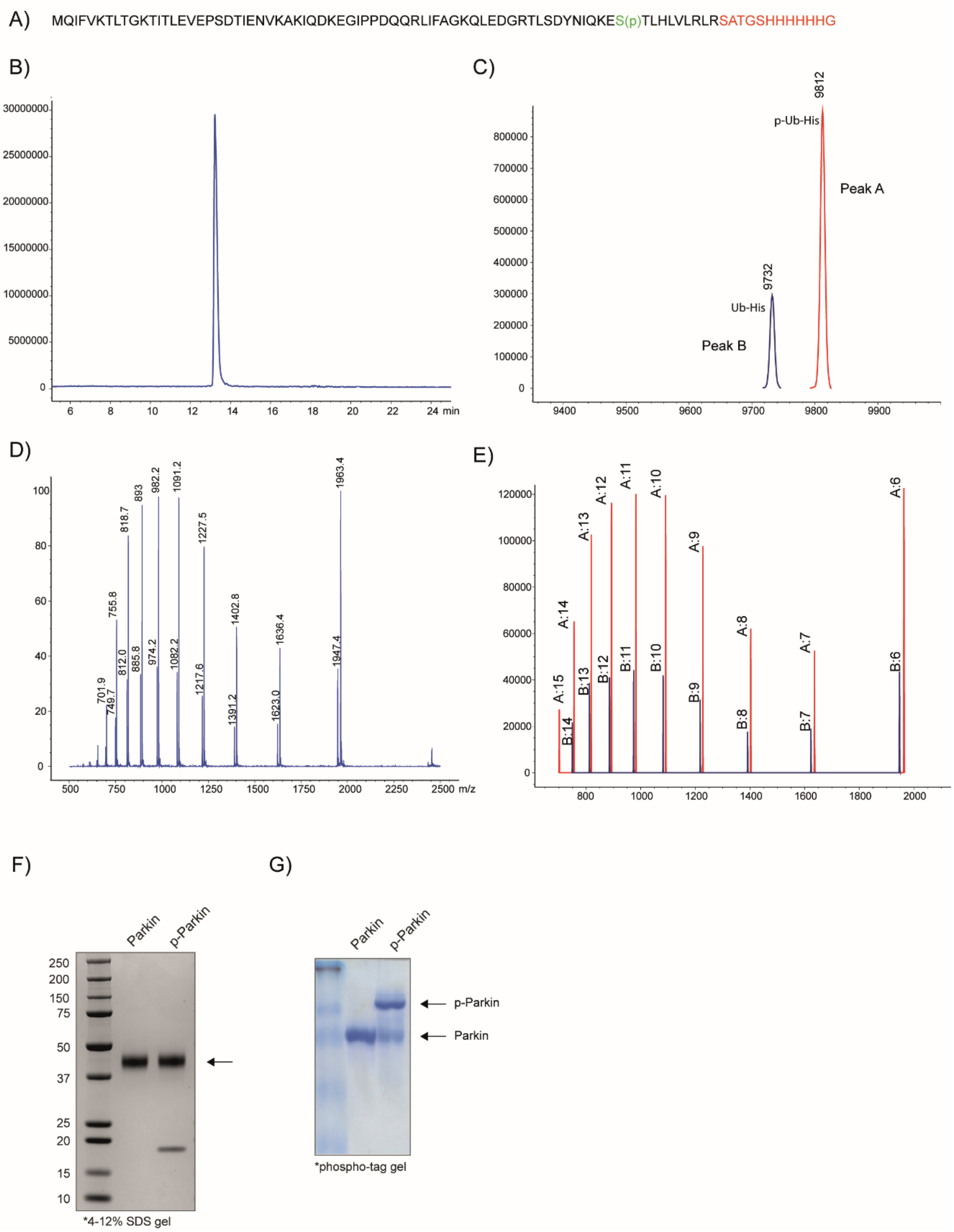
A)
MQIFVKTLTGKTITLEVEPSDTIENVKAKIQDKEGIPPDQQRLIFAGKQLEDGRTLSDYNIQKES(p)TLHLVLRLRSATGSHHHHHHG
B)
min
C)
9812
p-Ub-His
Peak A
9732
Ub-His
Peak B
D)
1963.4
1091.2
982.2
893
818.7
1227.5
755.8
1402.8
1636.4
812.0
885.8
974.2
1082.2
1947.4
701.9
749.7
1217.6
1391.2
1623.0
m/z
E)
A:15
A:14
A:13
A:12
A:11
A:10
A:9
A:8
A:7
A:6
B:14
B:13
B:12
B:11
B:10
B:9
B:8
B:7
B:6
F)
Parkin
p-Parkin
*4-12% SDS gel
G)
Parkin
p-Parkin
p-Parkin
Parkin
*phospho-tag gel

Sup Figure 1|His tagged Phosphorylated Ubiquitin (p-Ub-His), Parkin and pParkin Quality Control (related to Figure 2). (A) p-Ub-His protein sequence: serine 65 (green) and C-terminal 6His tag (red). p-Ub-His Quality Control (QC) performed via Liquid Chromatography - Mass Spectrometry (LC-MS) analysis. (B) LC-MS chromatogram of p-Ub-His. (C) p-Ub-His MS components: 9812 *m/z* peak, corresponding to the p-Ub-His expected *m/z*, and 9732 *m/z* peak corresponding to the remaining not phosphorylated counterpart. Purity level have been considered into experimental calculations. (D) p-Ub-His QC Mass Spectrum. (E) p-Ub-His QC Deconvoluted Ion Set. (F) Parkin and p-Parkin purity check by SDS-page. (G) Parkin phosphorylation efficiency: phospho-tag gels indicates that p-Parkin is about 70% pure. Arrow indicates main WT or p-Parkin species.

Sup Fig. 2

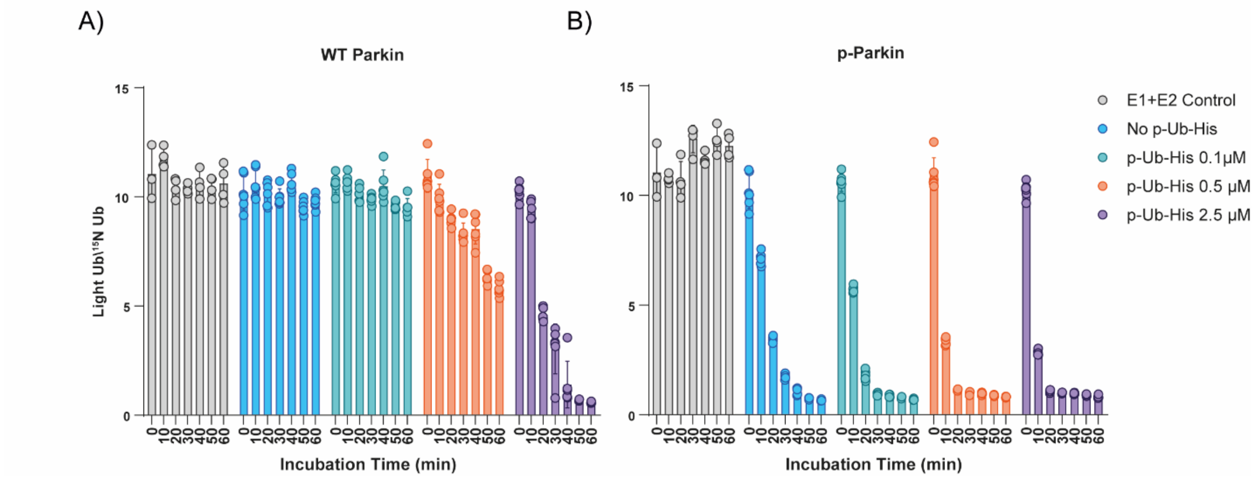

Sup. Figure 2| MALDI-TOF Ubiquitylation assay: Light/^{15}N ubiquitin ratio (related to Figure 2). Data shown before conversion into Residual Activity%. (A) WT Parkin MALDI-TOF autoubiquitylation assay: stable level of light/^{15}N Ub in the E1+E2 control indicates no consumption of ubiquitin in presence of the E1 activating enzyme and UBE2L3 conjugating enzyme only. (B) Corresponding dataset for p-Parkin MALDI-TOF autoubiquitylation assay. Data are represented as mean ± SD.

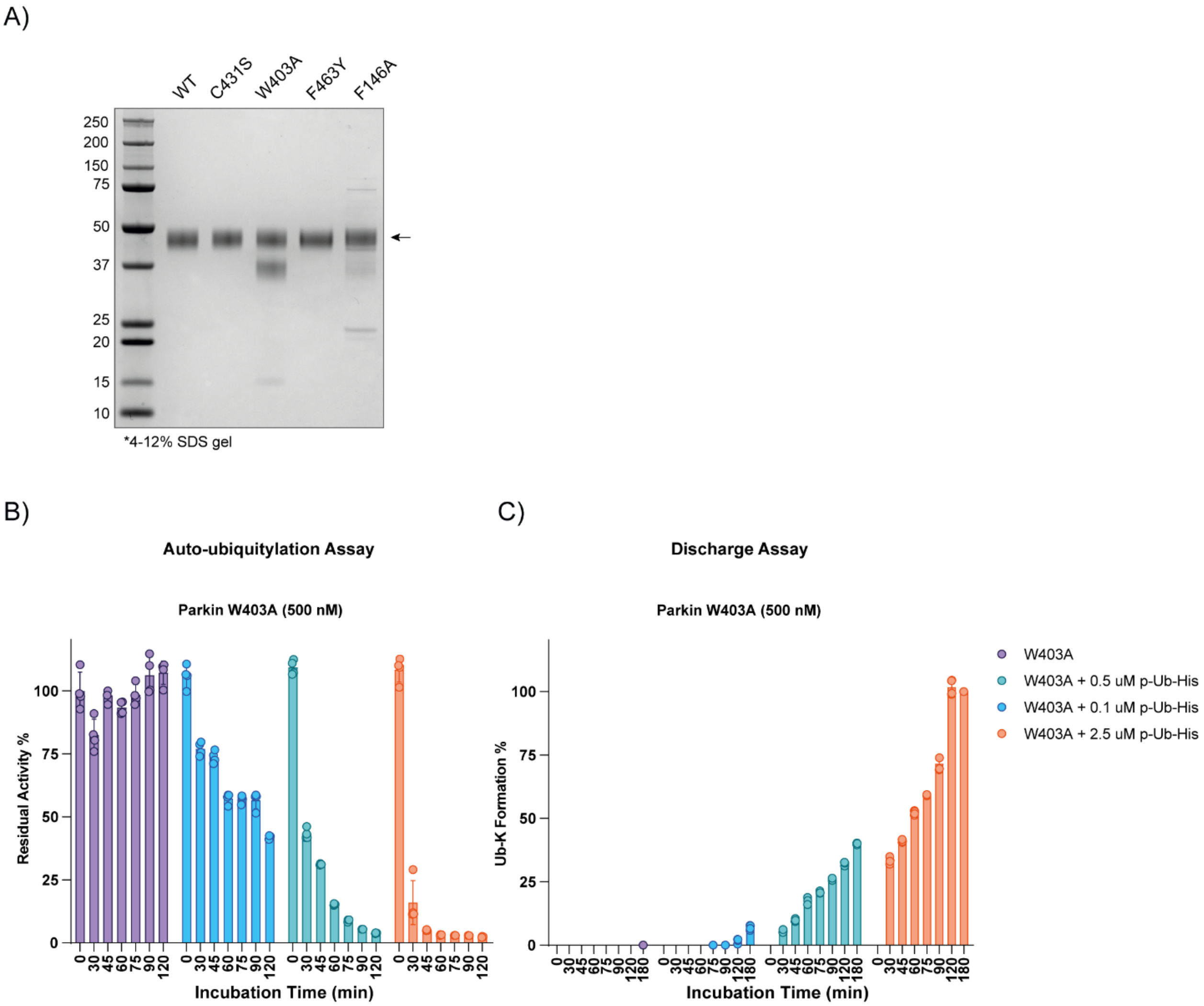

Sup. Figure 3| Parkin activating mutants purity check by SDS-page and activity at 500 nM (related to Figure 3). (A) Parkin WT, C431S, W403A, F463A and F146A were expressed and purified as indicated in Material and Methods. Relative quantification (main band indicated by the arrow) was implemented to account for the presence of impurities. (B) Activity of Parkin W403A was assessed at 500nM via MALDI-TOF autoubiquitylation assay. (C) Activity of Parkin W403A was assessed at 500nM via MALDI-TOF discharge assay. No background activity was detected when testing Parkin W403A at the final concentration of 500nM in absence of p-Ub-His. Indicated amount of p-Ub-His efficiently activated Parkin W403A. Data are represented as mean ± SD

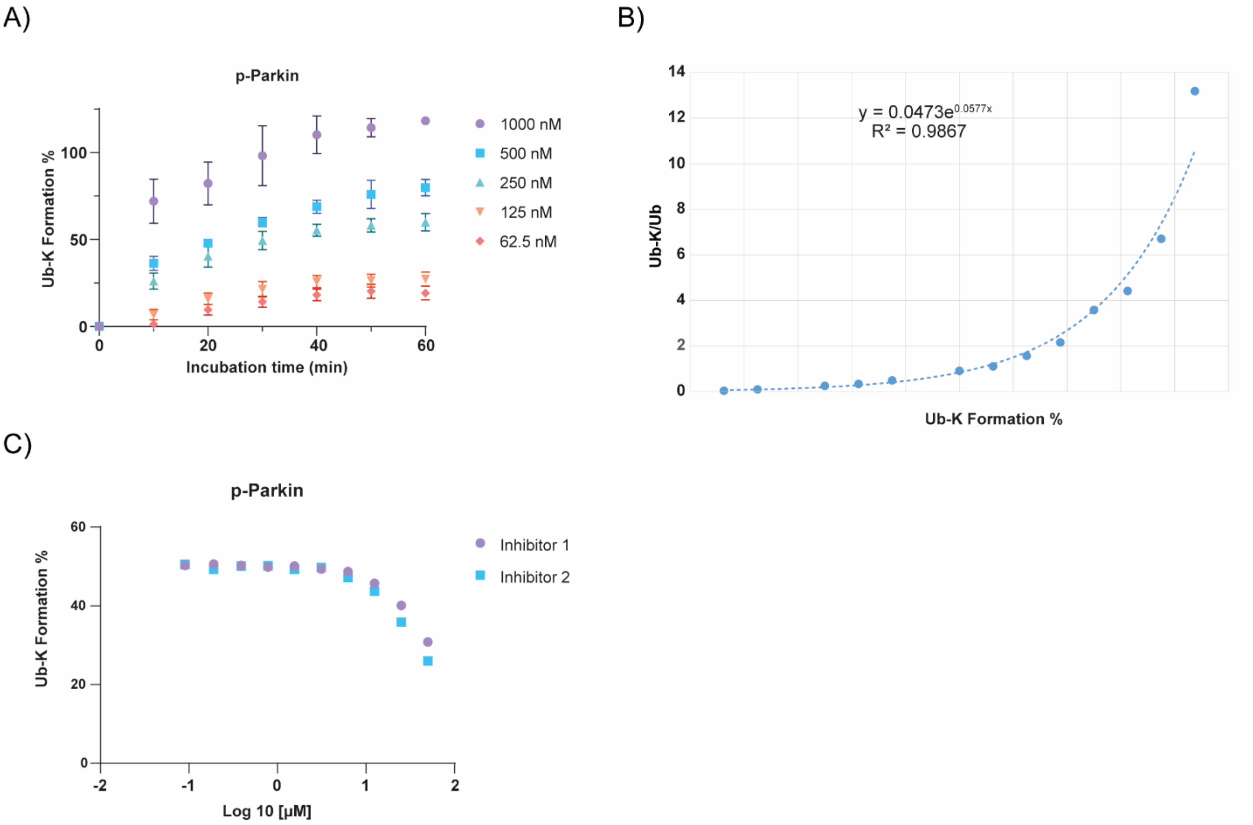

Sup. Figure 4| Identification of HTS conditions for p-Parkin MALDI-TOF discharge assay (related to Figure 4). (A) p-Parkin was incubated at 25° at the indicated at concentrations, and Ub-K formation was followed over a time course experiment. Data are represented as mean ± SD. (B) Linearity curve for Ub-K formation %. Known amounts of Ub (substrate) and Ub-K (product) were mixed and analysed by MALDI-TOF/MS. Resulting curve and associated exponential equation was employed to translate Ub-K/Ub peak area ratio into Ub-K Formation%. Data are represented as average of two datapoints. (C) Validation of two p-Parkin inhibitors identified via HTS. Compound 1 and compound 2 were tested at the indicated concentrations. Both compound 1 and compound 2 reduced p-Parkin activity at 10 μM, 25 μM and 50 μM. Data are represented as average of two datapoints.

Sup Fig. 5

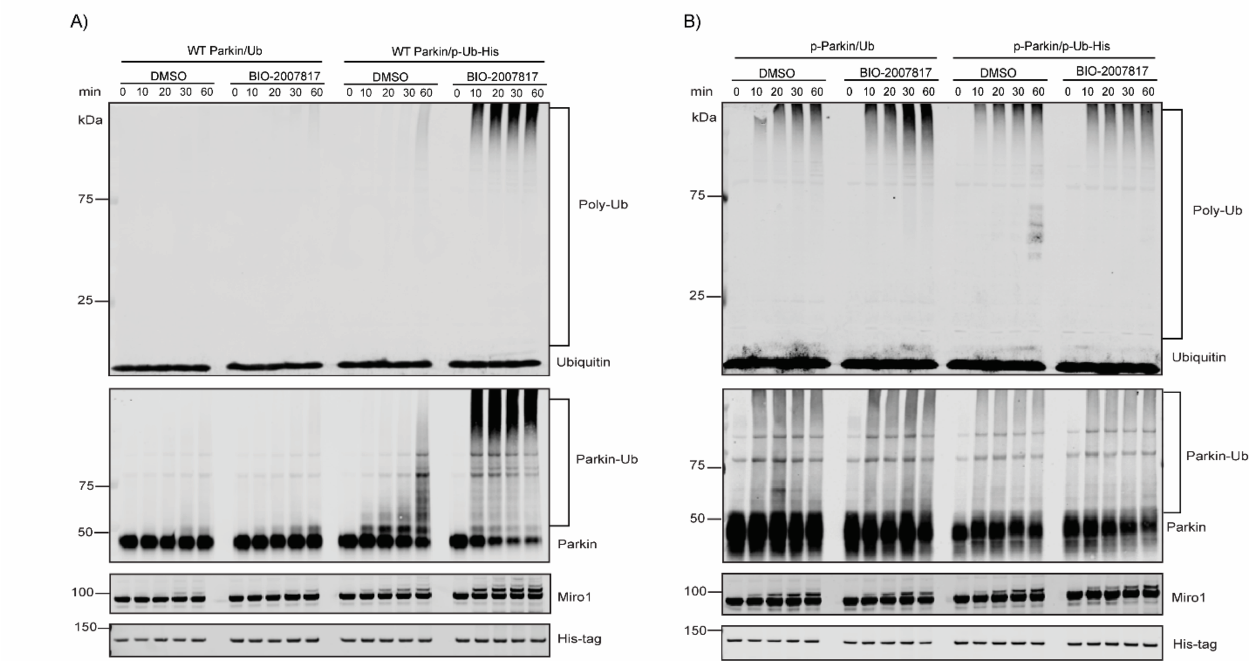

Sup. Fig. 5| Validation of BIO-2007817 as WT and p-Parkin activators via Western Blot analysis (related to Figure 4). (A) WT Parkin was tested in presence of BIO2007817 (100 µM) or DMSO (Control) with ubiquitin only (Ub) and with Ub + stoichiometric amount of p-Ub-His (relative to WT or p-Parkin). The reaction was stopped at the indicated time points and samples were run on SDS-page. Parkin and Miro1 ubiquitylation species were detected by Western Blot. (B) Similar to panel A, p-Parkin was tested in presence of BIO2007817 (100 µM) or DMSO (Control) with ubiquitin only (Ub) and with Ub + stoichiometric amount of p-Ub-His (relative to p-Parkin).
